# H3K36me3 modification by SETD2 is essential for *Col11a2* and *Sema3e* transcription to maintain dentinogenesis in mice

**DOI:** 10.1242/dev.204352

**Published:** 2025-07-14

**Authors:** Jiaxin Niu, Jing Fu, Hao Feng, Jiahao Han, Zhi Chen, Guobin Yang, Guohua Yuan

**Affiliations:** ^1^ State Key Laboratory of Oral & Maxillofacial Reconstruction and Regeneration, Key Laboratory of Oral Biomedicine Ministry of Education, Hubei Key Laboratory of Stomatology, School & Hospital of Stomatology, Wuhan University, Wuhan 430079, China; ^2^ Frontier Science Center for Immunology and Metabolism, Wuhan University, Wuhan 430079, China; ^3^ Hubei Provincial Key Laboratory of Developmentally Originated Disease, Wuhan 430071, China; ^4^ Pediatric Dentistry, Nanjing Stomatology Hospital, Medical School of Nanjing University, 30 Zhongyang Road, Nanjing 210008, China

**Keywords:** Odontoblast differentiation, Dentinogenesis, SET domain containing 2, Trimethylation of histone H3 at lysine 36, Histone methylation

## Abstract

Dentin is a major mineralized component of teeth generated by odontoblasts. Several types of histone methylation have been reported to play important roles in odontoblast differentiation and dentinogenesis. However, the role of methylation on histone 3 at lysine 36 (H3K36) remains enigmatic. Here, we demonstrate high expression of SETD2, a methyltransferase catalyzing the trimethylation of H3K36 (H3K36me3), in the odontoblast layer. *In vitro* knockdown experiments and *in vivo* observations of two conditional knockout mouse models reveal that SETD2 is essential for odontoblast differentiation and dentinogenesis. Integrated analyses of RNA sequencing and spike-in CUT&Tag sequencing data show that SETD2 is crucial for both H3K36me3 occupancy at the loci of *Col11a2* and *Sema3e* and their transcription. Further experiments verify that COL11A2 and SEMA3E act upstream of AKT1 signaling, promoting odontoblastic differentiation. *In vitro* and *in vivo* activation of AKT1 using SC79 (an AKT activator) partially rescues the impaired odontoblast differentiation caused by *Setd2* knockdown or deficiency. Therefore, our findings indicate that H3K36me3 mediated by SETD2 is essential for dentinogenesis by regulating the expression of *Col11a2* and *Sema3e* and AKT1 signaling.

## Teaser

**Summary:** *Setd2* deprivation in odontoblasts reduces both H3K36me3 occupancy at the loci of *Sema3e* and *Col11a2* and transcription of these two genes, leading to impaired AKT1 activation and dentin developmental defects.

## INTRODUCTION

Dentin is a major mineralized component of teeth. It has crucial functions in supporting the outer enamel and cementum as well as protecting the inner dental pulp (Goldberg et al., 2011). Odontoblasts are responsible for dentinogenesis. They are differentiated from the peripheral dental mesenchymal cells, and secrete extracellular matrix containing collagenous and non-collagenous proteins to form the predentin. Collagen fibers, mainly composed of type I collagen, form the structural framework for the subsequent deposition of calcium and phosphorus, thereby shaping the dentin. Non-collagenous proteins, including dentin sialophosphoprotein (DSPP) and dentin matrix protein 1 (DMP1), are crucial for mineralization and transformation of predentin into dentin (Bleicher, 2014; Butler, 1995).

Both genetic and epigenetic mechanisms are involved in regulating the complex process of odontoblast differentiation and dentinogenesis (Lin et al., 2018; Yu and Klein, 2020). Histone methylation is a crucial epigenetic feature that primarily occurs on lysine and arginine residues located at the termini of histone H3 and H4. Histone methyltransferases (HMTs), serving as ‘writers’, add methyl groups to histones, whereas histone demethylases, acting as ‘erasers’, remove these methyl groups (Jambhekar et al., 2019). Several HMTs or histone demethylases have been reported to regulate odontoblast differentiation by modifying different types of histone methylation. For example, euchromatic histone lysine N-methyltransferase 2 (EHMT2) catalyzes the mono- and dimethylation of histone H3 at lysine 9 (H3K9). Inactivation of *Ehmt2* in tooth mesenchyme leads to defects in odontoblast differentiation, characterized by shorter cellular processes and reduced expression of DSPP and DMP1 (Kamiunten et al., 2017). Lysine demethylase 5A (KDM5A), an eraser of di- and trimethylation of histone H3 at lysine 4 (H3K4me2/3), has been found to inhibit odontoblastic differentiation of human dental pulp cells by removing the activating H3K4me3 marks from the promoters of specific odontoblastic marker genes (Li et al., 2020). Enhancer of zeste homolog 2 (EZH2), a key catalytic subunit of the polycomb repressor complex 2 (PRC2), can catalyze the tri-methylation of histone H3 at lysine 27 (H3K27me3) to silence gene expression. An *in vitro* study has shown that EZH2-mediated H3K27me3 on the β-catenin promoter inhibits the expression of β-catenin to suppress odontoblastic differentiation of human dental pulp cells (Li et al., 2018). As an eraser of H3K27me3, lysine-specific demethylase 6B (KDM6B) plays a facilitating role in odontoblastic differentiation through the removal of the H3K27me3 marks from the bone morphogenic protein 2 (*Bmp2*) promoter (Xu et al., 2013). Interestingly, the loss of *Ezh2* in the dental mesenchyme of mice does not affect odontoblast differentiation (Jing et al., 2019). All the above studies have demonstrated the roles of different histone methylation modifications in odontoblast differentiation. Methylation of lysine 36 of histone H3 (H3K36) is closely related to various developmental processes (Janssen and Lorincz, 2022). However, there is no report regarding the involvement of methylation on H3K36 in odontoblast differentiation.

Methylation of H3K36 includes mono-, di- and trimethylation (H3K36me1, H3K36me2 and H3K36me3). Several HMTs, such as nuclear receptor binding nuclear receptor-binding  SET domain protein 1-3 (NSD1-3), ASH1like histone lysine methyltransferase (ASH1l), SET and MYND domain containing 2 (SMYD2) and SET domain and mariner transposase fusion gene (SETMAR) are responsible for catalyzing H3K36me1/2, while SET and MYND domain containing 5 (SMYD5) and SETD2 catalyze H3K36me3 (Michalak et al., 2019). H3K36 methylation is essential for the development of various tissues and organs (Janssen and Lorincz, 2022). H3K36 methylation is disrupted in mice carrying an H3K36M mutation and this mouse model exhibits differentiation defects during adipogenesis and myogenesis (Brumbaugh et al., 2019). The absence of NSD1, which catalyzes H3K36me1 and H3K36me2, has been shown to disrupt *de novo* DNA methylation in the male germline of mice, resulting in severe defects in spermatogenesis (Shirane et al., 2020). Variants or haploinsufficiency of *NSD1* cause Sotos syndrome or Weaver syndrome in humans with the phenotype of overgrowth (Douglas et al., 2003; Rio et al., 2003). Loss-of-function mutations in NSD2 lead to mild growth delay and prenatal-onset growth retardation, indicating a non-redundant or even opposite role of NSD2 to NSD1 (Zanoni et al., 2021). *Setd2*-deficient mice die at embryonic day (E) 10.5-E11.5 due to defects in vasculogenesis and angiogenesis (Hu et al., 2010). Deletion of *Setd2* in the primordial follicle results in oocyte maturation defects and subsequent one-cell arrest after fertilization (Xu et al., 2019). However, it is not known whether H3K36 methylation affects odontoblast differentiation.

In this study, we compared the mRNA levels of all commonly known HMTs that catalyze H3K36 methylation during the odontoblastic differentiation of mouse dental papilla cells (mDPCs) and found that the expression of *Setd2* was the highest at all tested time points. We therefore investigated the role of SETD2 in odontoblast differentiation using *in vitro* and *in vivo* experiments, both of which suggested a facilitating role of SETD2-mediated H3K36me3 on odontoblast differentiation. *Setd2* loss reduced H3K36me3 occupancy and transcription of *Col11a2* and *Sema3e*. Further experiments revealed that the downregulated COL11A2 and SEMA3E impaired AKT1 activation, leading to suppressed odontoblast differentiation and dentin formation.

## RESULTS

### Strong SETD2 and H3K36me3 signals are present during odontoblast differentiation

**Fig. 1.**
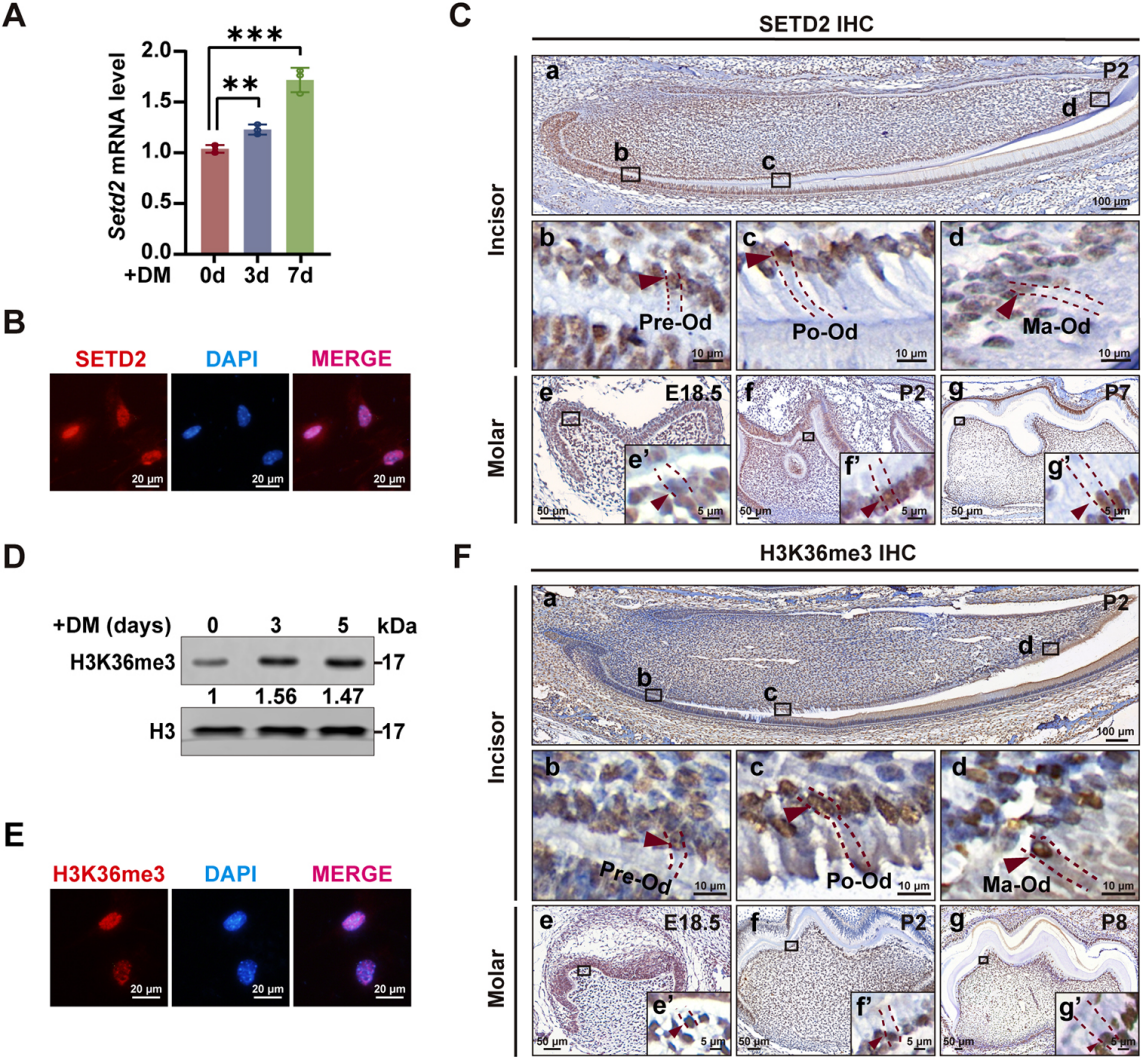
**Distribution pattern of SETD2 and H3K36me3 during the odontoblast differentiation.** (A) The mRNA level of *Setd2* in mDPCs cultured with differentiation medium (DM) for 0, 3 and 7 days was examined by RT-qPCR. Quantification data are presented as mean±s.d. and analyzed by one-way ANOVA with Tukey's post-test (*n*=3). **P<0.01; ***P<0.001. (B) Immunofluorescence (IF) showing the nuclear localization of SETD2 in mouse dental papilla cells (mDPCs) (*n*=3). (C) Immunohistochemistry (IHC) showing strong signals for SETD2 in the nucleus of the odontoblast layer from mouse incisors at P2 and from mouse molars at E18.5, P2 and P7. Panels b-d show higher magnifications of the corresponding rectangles in a. The magnified views in e′-g′ correspond to the rectangles in e-g, respectively (E18.5, *n*=3; P2, *n*=6; P7, *n*=3). (D) Western blot showing the presence of H3K36me3 in mDPCs cultured with DM for 0, 3 and 5 days. Histone H3 was used as the loading control. (E) IF showing the nuclear localization of H3K36me3 in mDPCs (*n*=3). (F) IHC showing nuclear signals for H3K36me3 in the odontoblast layer of mouse incisors at P2 and of mouse molars at E18.5, P2 and P8. Panels b-d show higher magnifications of the corresponding rectangles in a. The magnified views in e′-g′ correspond to the rectangles in e-g, respectively. Red arrows in C and F point to the positive signals in the nucleus. Dashed lines in C and F mark the boundaries of the odontoblasts at different differentiation stages (E18.5, *n*=3; P2, *n*=6; P8, *n*=3). Ma-Od, mature odontoblasts; Po-Od, polarizing odontoblasts; Pre-Od, preodontoblasts.

To unveil a possible role of H3K36 methylation during odontoblast differentiation, we sought to screen the expression of all HMTs that regulate H3K36 methylation, including *Nsd1-3*, *Ash1l*, *Smyd2*, *Setmar*, *Smyd5* and *Setd2*, in a cell culture system of odontoblastic differentiation using RT-qPCR. The results showed that the expression of *Setd2* was the highest at all time points among all the tested HMTs (Fig. S1A). Analysis of the RT-qPCR results showed an increasing trend of *Setd2* expression during the odontoblastic differentiation of mDPCs (Fig. 1A). Immunofluorescence staining (IF) revealed a nuclear distribution of SETD2 in mDPCs (Fig. 1B). Therefore, we focused on SETD2 in the following experiments. To examine the spatiotemporal expression pattern of SETD2, immunohistochemistry (IHC) was performed in mouse incisors on postnatal day (P) 2, which allows the observation of all differentiation stages of odontoblasts in a single slice (Fig. 1Ca-Cd). The results showed that SETD2 was highly expressed in the nucleus of preodontoblasts (Fig. 1Cb), polarizing odontoblasts (Fig. 1Cc) and mature odontoblasts (Fig. 1Cd). To confirm the expression of SETD2 during odontoblast differentiation, IHC of SETD2 was performed in mouse molars at different developmental stages. Strong SETD2 signals were seen in the odontoblast layer of mouse molars at E18.5, P2 and P7 (Fig. 1Ce-Cg). No positive signal was observed in the negative control for IHC (Fig. S1B).

Since SETD2 catalyzes H3K36me3 (Michalak et al., 2019), we next examined the localization of H3K36me3 in cultured mDPCs and whole tooth slices. The results showed that H3K36me3 was present during the odontoblastic differentiation of mDPCs and localized in the nucleus of mDPCs as shown by western blot and IF (Fig. 1D,E). IHC showed nuclear localization of H3K36me3 in preodontoblasts, polarizing odontoblasts and mature odontoblasts of incisors at P2 (Fig. 1Fa-Fd), which is similar to the expression pattern of SETD2 in incisors. IHC also showed the distribution of H3K36me3 in the odontoblast layer of mouse molars at E18.5, P2 and P8 (Fig. 1Fe-Fg). Some other cell types, such as ameloblasts and dental papilla cells, were also positive for SETD2 and H3K36me3 in the whole tooth slices (Fig. 1C,F).

### SETD2 promotes odontoblast differentiation and dentinogenesis

**Fig. 2.**
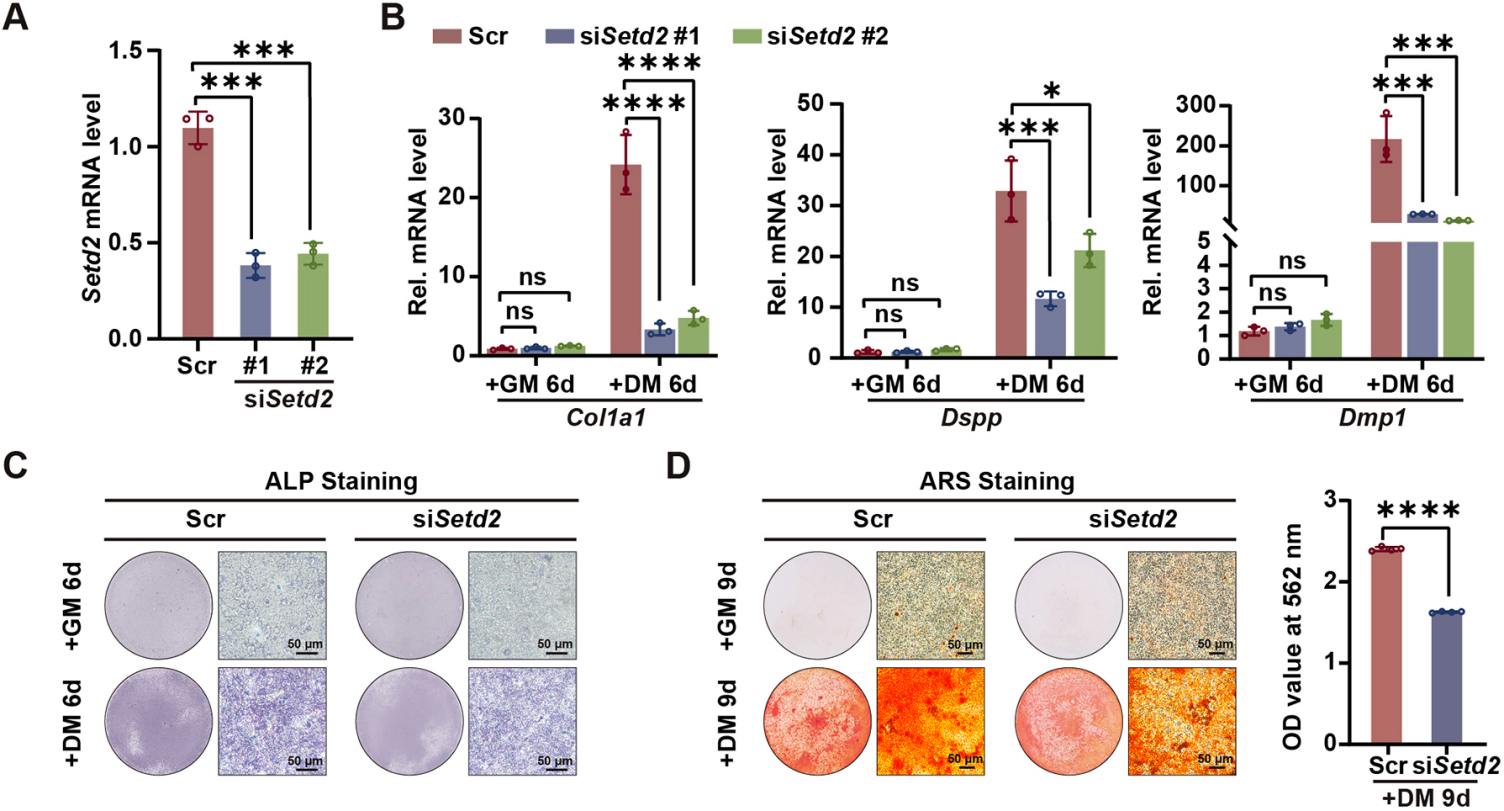
**SETD2 promotes the odontoblastic differentiation of mDPCs.** (A) Knockdown efficiency of two different siRNAs targeting *Setd2* in mDPCs detected by RT-qPCR. The quantification data are presented as mean±s.d. and were analyzed by one-way ANOVA with Tukey's post-test (*n*=3). (B) The mRNA levels of odontoblastic marker genes (*Col1a1*, *Dspp* and *Dmp1*) were assessed by RT-qPCR. mDPCs were cultured with growth medium (GM) or DM for 6 days following transfection with two *Setd2* siRNAs or scramble siRNA. The quantification data are presented as mean±s.d. and were analyzed by one-way ANOVA with Tukey's post-test (*n*=3). (C) Alkaline phosphatase (ALP) staining shows the activity of ALP in mDPCs transfected with scramble or *Setd2* siRNA and cultured with GM or DM for 6 days (*n*=3). (D) Alizarin Red S (ARS) staining shows mineralized nodule formation in mDPCs transfected with scramble or *Setd2* siRNA and cultured with GM or DM for 9 days. Stained cells were washed with 10% cetylpyridinium chloride, and the extracted dye was quantified by measuring the optical density (OD) values at 562 nm. The quantification data are presented as mean±s.d. and were analyzed by two-tailed unpaired Student's *t*-test (*n*=5). DM, differentiation medium; GM, growth medium; ns, not significant; Scr, scramble; si, siRNA. *P<0.05; ***P<0.001; ****P<0.0001.

To explore the role of SETD2 during odontoblast differentiation, *Setd2* was knocked down in mDPCs using two different siRNAs, which gave rise to ∼60% reduction of *Setd2* expression (Fig. 2A). RT-qPCR results showed that the expression levels of the odontoblastic marker genes *Col1a1*, *Dspp* and *Dmp1* were notably reduced in the *Setd2* knockdown group compared with those in the control group after differentiation induction (Fig. 2B). Alkaline phosphatase (ALP) staining and Alizarin Red S (ARS) staining showed that both ALP activity and mineralized nodule formation were obviously impaired in *Setd2* knockdown group (Fig. 2C,D).

To confirm the function of SETD2 *in vivo*, we obtained *Ubc-*CreERT2*;Setd2*^fl/fl^ mice, which were intraperitoneally injected with tamoxifen at P4 to induce universal *Setd2* inactivation and harvested at P14. IHC staining demonstrated the successful deletion of *Setd2* in this mouse model (Fig. S2A). Micro-computed tomography (μ-CT) of the mandibular first molars revealed a notable reduction in the thickness of the dentin in both the crown and root regions of *Ubc-*CreERT2*;Setd2*^fl/fl^ mice at P14. Quantitative analysis through three-dimensional reconstruction further revealed that *Setd2* deficiency resulted in decreased dentin volume (Fig. S2B). At P4, the root development of the mandibular first molars initiates, and the odontoblasts in the crown area have fully differentiated (Li et al., 2017). Hematoxylin and Eosin (HE) staining showed that the root area of *Ubc-*CreERT2*;Setd2*^fl/fl^ mice at P14 displayed impaired odontoblast differentiation characterized by shorter cell processes, decreased dentinogenesis characterized by reduced dentin widths, and increased proportions of non-mineralized predentin relative to total dentin, indicating that both differentiation and secretion function of odontoblasts are diminished (Fig. S2Ca,Cc,Ce). Meanwhile, in the crown area, the thickness of the crown dentin was also reduced, suggesting that inactivation of *Setd2* in differentiated odontoblasts impairs their secretion ability (Fig. S2Cb,Cd,Ce). These findings suggest that the widespread deletion of *Setd2* in *Ubc*-CreERT2*;Setd2*^fl/fl^ leads to defects in odontoblast differentiation and secretion.

**Fig. 3.**
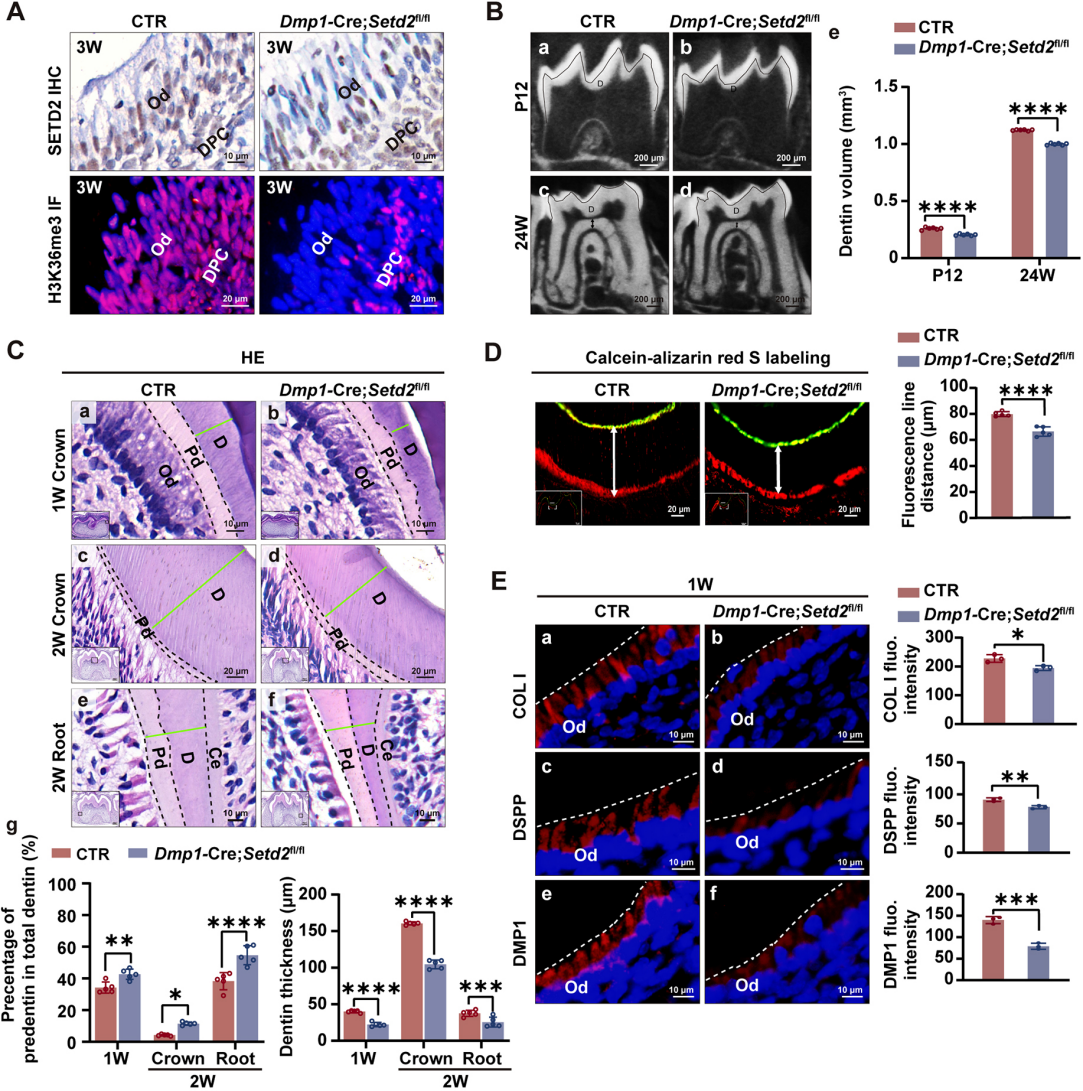
**SETD2 is required for odontoblast differentiation and dentinogenesis.** (A) The expression of SETD2 is absent in the odontoblast layer of the mouse molars from *Dmp1*-Cre*;Setd2*^fl/fl^ mice but present in that from the control (CTR) mice, as shown by IHC. In contrast to control mice, IF shows a lack of H3K36me3 in the odontoblast layer of the mouse molars from *Dmp1*-Cre*;Setd2*^fl/fl^ mice (*n*=3). (B) Representative μ-CT images of the first mandibular molars show decreased dentin widths in *Dmp1*-Cre*;Setd2*^fl/fl^ mice compared with control mice at P12 and 24W (a-d). Double-headed arrows show the dentin thickness at the pulp chamber floor. Quantitative analyses after three-dimensional reconstruction show reduced dentin volume in *Dmp1*-Cre*;Setd2*^fl/fl^ mice at P12 and 24W (e) (P12, *n*=6/genotype; 24W, *n*=6/genotype). (C) HE staining of mouse molars shows shorter cellular processes of odontoblasts and thinner dentin thickness in *Dmp1*-Cre*;Setd2*^fl/fl^ mice (b,d,f) compared with control mice (a,c,e) at 1W and 2W. The thickness of the predentin and dentin was measured (green lines) in one section out of every five consecutive sections of five HE-stained samples, and the quantitative analysis is shown in g (1W, *n*=5/genotype; 2W, *n*=5/genotype). (D) Calcein-Alizarin Red S double labeling was performed to compare the dentin deposition rate between control and *Dmp1*-Cre*;Setd2*^fl/fl^ mice (*n*=5/genotype). Double-headed arrows show the measurement taken for quantification of dentin deposition. Insets show the areas that the higher magnifications are from. (E) IF and quantitative analysis of fluorescence intensity of odontoblast markers (collagen I, DSPP and DMP1) in the odontoblast layer of mouse molars. The quantification data are presented as mean±s.d. and were analyzed by two-tailed unpaired Student's *t*-test (*n*=3). Ce, cementum; CTR, control mice; D, dentin; DPC, dental papilla cell; HE, Hematoxylin and Eosin staining; Od, odontoblasts; Pd, predentin. *P<0.05; **P<0.01; ***P<0.001; ****P<0.0001.

*Dmp1*-Cre has been used to selectively target the odontoblast layer *in vivo* (Lu et al., 2007; Zheng et al., 2022). Therefore, we generated *Dmp1*-Cre*;Setd2*^fl/fl^ mice to confirm the role of *Setd2* in the odontoblast layer. Immunostaining showed that the distribution of SETD2 and H3K36me3 disappeared in the odontoblasts but remained in the dental pulp cells of *Dmp1*-Cre*;Setd2*^fl/fl^ mice at 3 weeks of age (3W), indicating an efficient and specific deletion of *Setd2* in the odontoblast layer (Fig. 3A). μ-CT of the mandibular first molars revealed a notable reduction of dentin thickness in *Dmp1*-Cre*;Setd2*^fl/fl^ mice at P12 and 24W. Quantitative analysis revealed decreased dentin volume in *Dmp1*-Cre*;Setd2*^fl/fl^ mice (Fig. 3B). HE staining revealed impaired odontoblast differentiation characterized by shorter cell processes, reduced dentin widths as well as increased proportions of non-mineralized predentin relative to total dentin in *Dmp1*-Cre*;Setd2*^fl/fl^ mice at both 1W and 2W. Additionally, at 1W, an irregular mineralization front between predentin and mineralized dentin was observed (Fig. 3C). To determine whether *Setd2* deficiency influenced the rate of dentin deposition, double fluorescence labeling using Calcein and ARS was applied. The results showed an evident decrease in the distance between the Calcein and ARS lines in *Dmp1*-Cre*;Setd2*^fl/fl^ mice (Fig. 3D). Meanwhile, the expression levels of the odontoblast markers collagen I, DSPP and DMP1 were reduced in the odontoblast layer of *Dmp1*-Cre*;Setd2*^fl/fl^ mice detected by IF staining (Fig. 3E, Fig. S3A). The above *in vitro* and *in vivo* findings collectively indicate that SETD2 plays a positive role in odontoblast differentiation and dentinogenesis.

### *Setd2* ablation decreases H3K36me3 occupancy and expression of *Col11a2* and *Sema3e* in odontoblasts

**Fig. 4.**
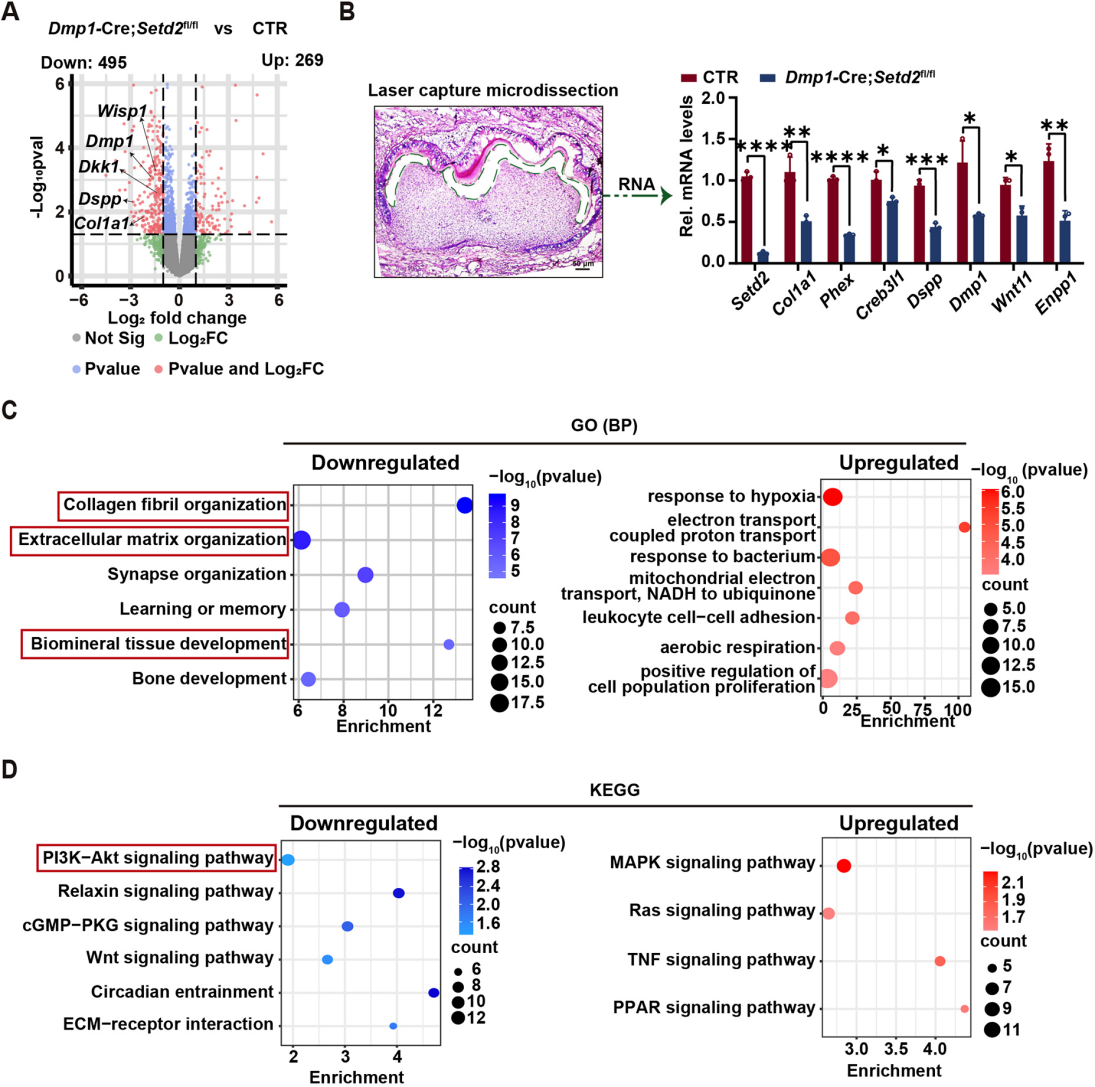
**RNA-seq analysis reveals differentially expressed genes upon *Setd2* ablation.** (A) Volcano plot showing up- and downregulated genes in the dental papilla cells from *Dmp1*-Cre*;Setd2*^fl/fl^ mice compared with control (*n*=3). (B) RNAs were extracted following laser-capture microdissection of the odontoblast layer from the molars of control and *Dmp1*-Cre*;Setd2*^fl/fl^ mice, followed by RT-qPCR to validate mRNA level changes observed in the RNA-seq data. The quantification data are presented as mean±s.d. and were analyzed by two-tailed unpaired Student's *t*-test (*n*=3). *P<0.05; **P<0.01; ***P<0.001; ****P<0.0001. (C) GO analysis revealed the biological process terms enriched by downregulated and upregulated genes in *Dmp1*-Cre*;Setd2*^fl/fl^ mice. (D) KEGG analysis revealed the pathway terms based on RNA-seq datasets for upregulated and downregulated genes in *Dmp1*-Cre*;Setd2*^fl/fl^ mice, and the items marked by red rectangles are the pathways of interest. CTR, control mice; cKO, *Dmp1*-Cre*;Setd2*^fl/fl^ mice.

To uncover the underlying mechanism by which SETD2 facilitates odontoblast differentiation, RNA sequencing (RNA-seq) was performed to compare gene expression profiles of dental mesenchymal cells from *Dmp1-*Cre*;Setd2*^fl/fl^ mice with those from control littermates. RNA-seq identified 764 significantly differentially expressed genes (fold change >1.5), including 269 upregulated and 495 downregulated genes in *Dmp1*-Cre*;Setd2*^fl/fl^ mice. Of note, consistent with the dentin defects observed in *Dmp1*-Cre*;Setd2*^fl/fl^ mice, some genes essential for odontoblast differentiation, *Dspp*, *Dmp1*, *Dkk1*, *Wisp1* (*Ccn4*) and *Sall1*, were significantly downregulated in *Dmp1-*Cre*;Setd2*^fl/fl^ mice (Fig. 4A, Fig. S4). To confirm the alterations in gene expression occurred in the odontoblast layer, laser-capture microdissection was performed to specifically collect the cells at the odontoblast layer of mouse molars at P3. Some genes that are crucial for odontoblast differentiation with downregulated expression in the RNA-seq data were re-examined by RT-qPCR. The results verified that all of the investigated genes, *Col1a1*, *Phex*, *Creb3l1*, *Dspp*, *Dmp1*, *Wnt11* and *Enpp1*, were significantly downregulated (Fig. 4B). Gene Ontology (GO) and Kyoto Encyclopedia of Genes and Genomes (KEGG) analysis were conducted on both upregulated and downregulated genes. GO analysis revealed enrichment of downregulated genes involved in collagen fibril organization, extracellular matrix organization and biomineral tissue development (Fig. 4C, Table S1). KEGG analysis of the RNA-seq data showed that downregulated genes in *Dmp1*-Cre*;Setd2*^fl/fl^ mice were enriched in the PI3K/AKT pathway (Fig. 4D, Table S2). Notably, previous studies have demonstrated that AKT signaling positively regulates odontoblast differentiation (Wang et al., 2023). Therefore, in subsequent experiments we focused on exploring the role of this pathway.

**Fig. 5.**
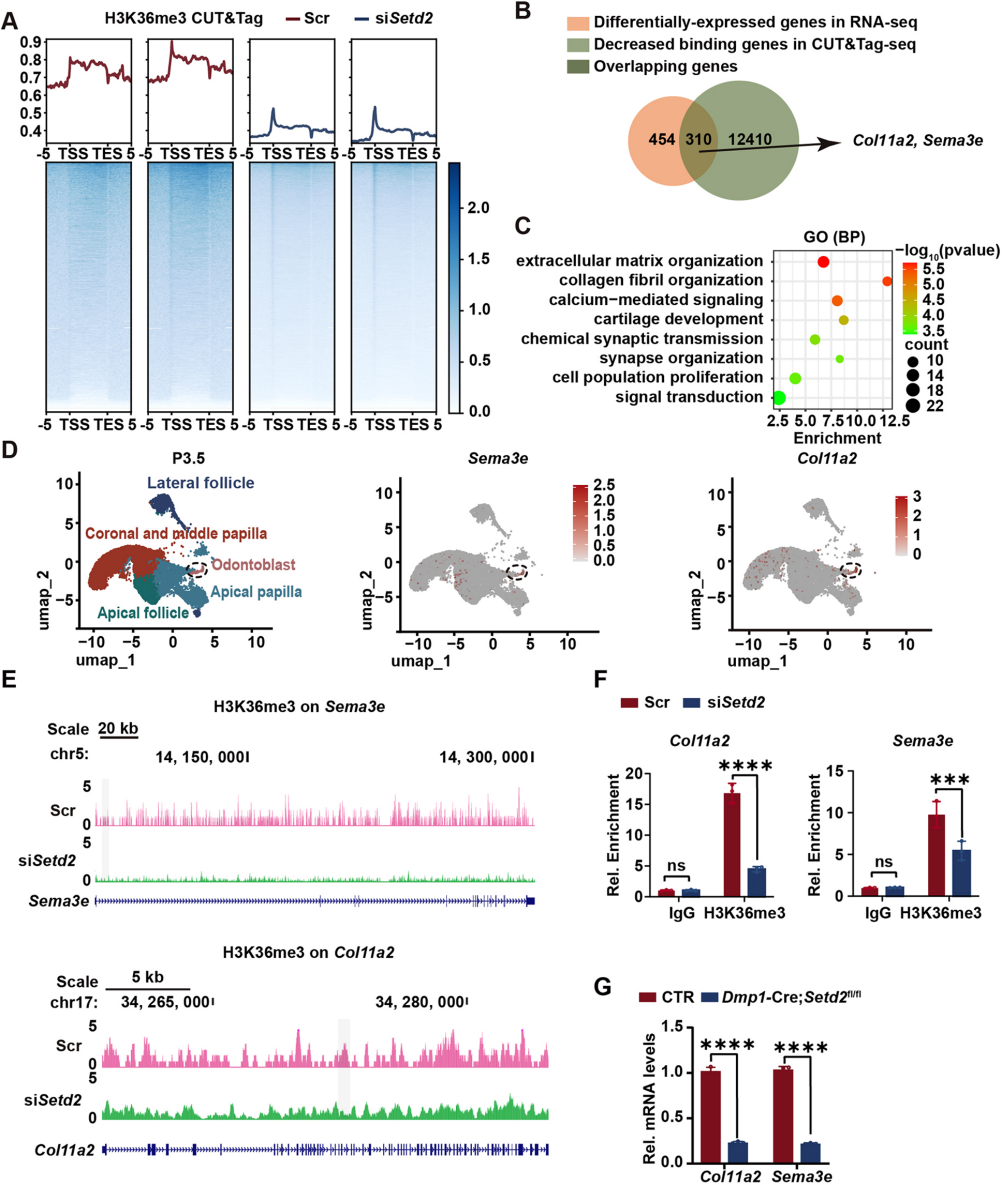
***Setd2* knockdown diminishes H3K36me3 occupancy at *Col11a2* and *Sema3e* loci, accompanied by their transcriptional downregulation in the odontoblasts.** (A) Heat maps showing the normalized read density of H3K36me3 CUT&Tag signals in the odontoblast-like cells with *Setd2* knockdown compared with control cells transfected with scramble siRNA spanning 5 kb upstream of the transcription start site (TSS) to 5 kb downstream of the transcription end site (TES) (*n*=2). (B) Venn diagram showing the number of genes with differential expression in RNA-seq, the number of genes with decreased occupancy of H3K36me3 in CUT&Tag-seq, and the overlapping genes (e.g. *Col11a2* and *Sema3e*). (C) GO analysis of the overlapping genes in B showing the enriched biological process terms. (D) Uniform manifold approximation and projection plots using the Jing et al. (2022) dataset illustrate cell clustering of P3.5 mouse molar cells and the presence of the transcripts for *Sema3e* and *Col11a2* in the odontoblast cluster. Dashed circles indicate the cluster of odontoblasts. (E) H3K36me3 CUT&Tag-seq results showing its peaks at the gene loci of *Col11a2* and *Sema3e* in the odontoblast-like cells with or without *Setd2* knockdown. (F) CUT&Tag-qPCR analysis of H3K36me3 occupancy at *Col11a2* and *Sema3e* gene loci, using isotype IgG as a negative control to assess non-specific binding. The quantification data are presented as mean±s.d. and were analyzed by two-tailed unpaired Student's *t*-test (*n*=3). (G) The odontoblasts in the molars of control and *Dmp1*-Cre*;Setd2*^fl/fl^ mice were collected using laser-capture microdissection. RNAs were extracted, followed by RT-qPCR to validate the changes in the mRNA levels of *Col11a2* and *Sema3e*. The quantification data are presented as mean±s.d. and were analyzed by two-tailed unpaired Student's *t*-test (*n*=3). CTR, control mice; ns, not significant; Scr, scramble. ***P<0.001; ****P<0.0001.

**Fig. 6.**
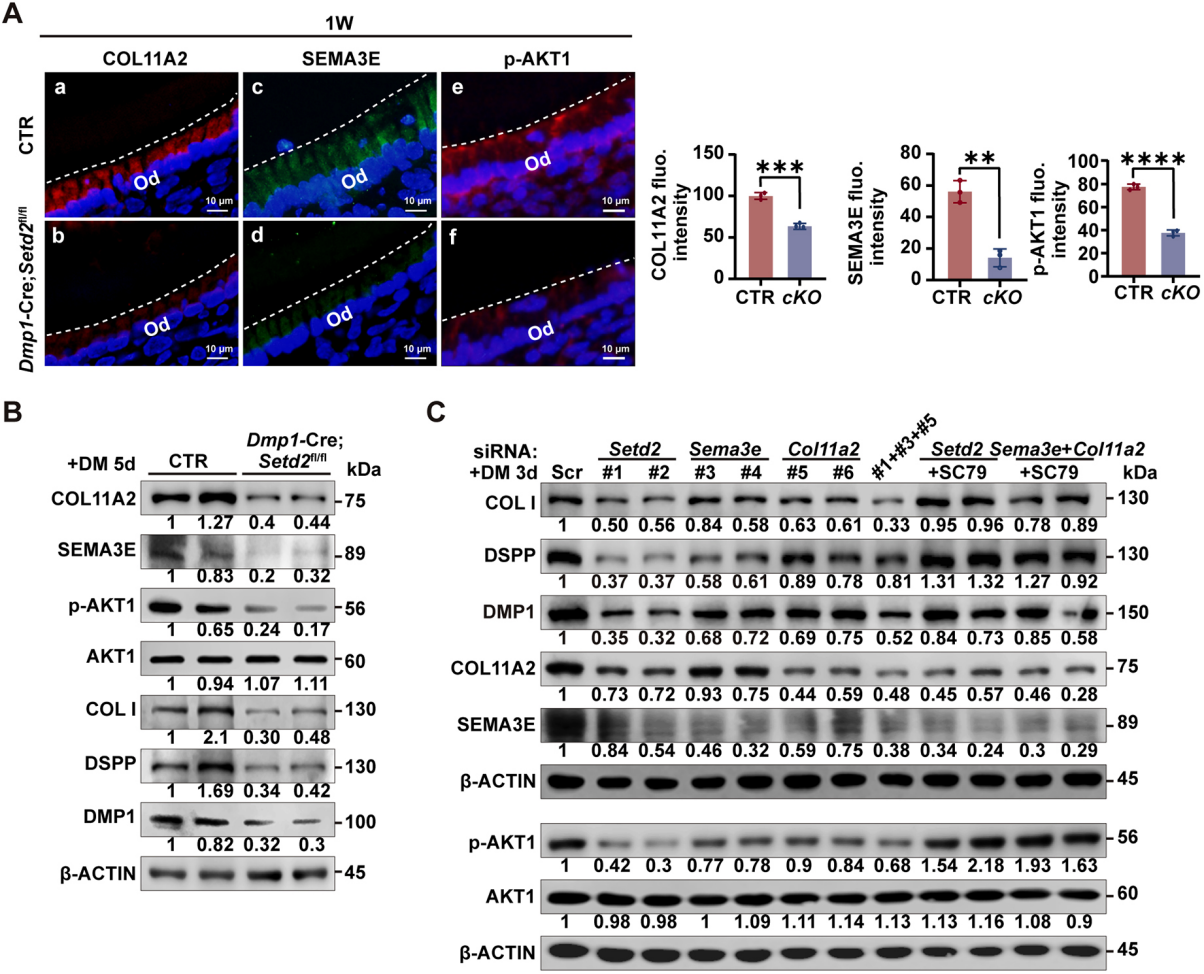
**SEMA3E and COL11A2 promote activation of AKT1, thus regulating odontoblast differentiation.** (A) IF and the quantitative analysis of the fluorescence intensity of COL11A2, SEMA3E and p-AKT1 in the odontoblast layer of mouse molars. The quantification data are presented as mean±s.d. and were analyzed by two-tailed unpaired Student's *t*-test (*n*=3). **P<0.01; ***P<0.001; ****P<0.0001. (B) mDPCs were obtained from control and *Dmp1*-Cre*;Setd2*^fl/fl^ mice and cultured in DM for 5 days, followed by western blot assays of COL11A2, SEMA3E, collagen I, DSPP, DMP1, AKT1 and p-AKT1. β-Actin was used as a loading control. (C) mDPCs were transfected with siRNAs targeting *Setd2*, *Sema3e* and *Col11a2* alone or in combination, followed by western blot analysis of collagen I, DSPP, DMP1, AKT1, p-AKT1, COL11A2 and SEMA3E. β-Actin served as a loading control. CTR, control mice; Scr, scramble.

Several studies indicate that H3K36me3 plays a role in the transcriptional regulatory process. For example, loss of SETD2-catalyzed H3K36me3 impairs osteogenic differentiation and enhances adipogenic differentiation of bone marrow mesenchymal stem cells (BMSCs), attributed to reduced RNA polymerase II (RNA POL II) occupancy on the *Lbp* gene locus which causes diminished transcriptional initiation and elongation of *Lbp* (Wang et al., 2018). Additionally, SETD2 catalyzes H3K36me3 at the distal promoter region of *Fgfr3* to regulate its transcription initiation, modulating FGF signaling and controlling the differentiation of embryonic stem cells into primitive endoderm (Zhang et al., 2014). Furthermore, H3K36me3, mediated by SETD2, recruits DNMT3B to prevent spurious transcription initiation, ensuring transcription fidelity (Neri et al., 2017). To identify the gene(s) directly regulated by SETD2-mediated H3K36me3 on a genome-wide scale, mDPCs were transfected with the *Setd2* or scramble siRNA and induced for odontoblastic differentiation for 5 days. RT-qPCR and western blot experiments showed reduced *Setd2* mRNA as well as decreased protein levels of SETD2 and H3K36me3, supporting an efficient knockdown of *Setd2* (Fig. S5). Subsequently, spike-in cleavage under targets and tagmentation (spike-in CUT&Tag) experiments were performed using an anti-H3K36me3 antibody, followed by next-generation sequencing. The results showed that the total H3K36me3 occupancy was clearly reduced in the odontoblast-like cells by *Setd2* knockdown (Fig. 5A, Fig. S6). We identified 310 genes with both reduced H3K36me3 occupancy and altered expression by integrating RNA-seq and CUT&Tag-seq data (Fig. 5B, Table S3). GO analysis showed that the overlapped genes were enriched in ‘extracellular matrix organization’, ‘collagen fibril organization’ and ‘calcium-mediated signaling’ (Fig. 5C, Table S4), which are consistent with the phenotype of dentin defects in *Dmp1*-Cre*;Setd2*^fl/fl^ mice. Among these genes, we noticed that *Sema3e* and *Col11a2*, which have been reported to play a role in the development of other organs (Avcin et al., 2008; Meadows et al., 2012; Melkoniemi et al., 2000; Tompson et al., 2012), exhibit specific expression in the odontoblasts (Fig. 5D). Spike-in CUT&Tag-seq analyses revealed that H3K36me3 levels on *Sema3e* and *Col11a2* were decreased upon *Setd2* knockdown (Fig. 5E). The reduction of H3K36me3 at specific peaks on both genes was further validated by CUT&Tag-qPCR (Fig. 5F). Meanwhile, the odontoblast layer was obtained using laser-capture microdissection, and RT-qPCR showed that *Col11a2* and *Sema3e* expression was significantly downregulated in the odontoblasts from *Dmp1*-Cre*;Setd2*^fl/fl^ mice (Fig. 5G). IF results indicated a reduction in the levels of COL11A2 and SEMA3E within the odontoblast layer of *Dmp1*-Cre*;Setd2*^fl/fl^ mice compared to control littermates at 1W (Fig. 6Aa-Ad). Additionally, mDPCs isolated from *Dmp1*-Cre*;Setd2*^fl/fl^ and control mice were cultured and subjected to odontoblastic differentiation. Subsequent western blot analysis further validated that the expression of COL11A2 and SEMA3E was significantly lower in *Setd2*-deficient odontoblastic cells (Fig. 6B). Taken together, these data indicate that *Col11a2* and *Sema3e* are downstream targets regulated by SETD2 in odontoblasts.

### Downregulated SEMA3E and COL11A2 in *Dmp1*-Cre*;Setd2*^fl/fl^ mice affect odontoblast differentiation via AKT1

Previous studies have demonstrated that both COL11A2 and SEMA3E can activate the PI3K/AKT signaling pathway (Agrawal et al., 2024; Hagihara et al., 2022). AKT comprises three isoforms: AKT1, AKT2 and AKT3. Analysis of previously published single-cell RNA-seq (scRNA-seq) data (Jing et al., 2022; GSE189381) showed that the expression of *Akt1* is the highest among these three AKT isoforms in the odontoblasts, whereas *Akt2* and *Akt3* are weakly expressed (Fig. S7A). IF results demonstrated a marked decrease in phosphorylated AKT1 (p-AKT1), the activated form of AKT1, within the odontoblast layer of *Dmp1*-Cre*;Setd2*^fl/fl^ mice to control littermates at 1W (Fig. 6Ae,Af). Additionally, mDPCs from *Dmp1*-Cre*;Setd2*^fl/fl^ and control littermate mice were obtained and induced for odontoblastic differentiation. Western blot assays showed reduced expression of odontoblast differentiation markers (collagen I, DSPP and DMP1) as well as diminished p-AKT1 level in the *Setd2*-deficient cells (Fig. 6B), reinforcing the association between *Setd2* deficiency and impaired AKT1 activation.

To ascertain the upstream and downstream relationship of p-AKT1 with SEMA3E and COL11A2 and to explore the roles of SEMA3E and COL11A2 in the odontoblastic differentiation of mDPCs, *Sema3e* and *Col11a2* were knocked down using small interfering RNAs (siRNAs) in mDPCs, which gave rise to an approximately 60% reduction of *Sema3e* and 70% reduction of *Col11a2* mRNAs as assessed by RT-qPCR (Fig. S7B). Western blot analysis revealed that knockdown of either *Sema3e* or *Col11a2* in mDPCs led to a reduction in the levels of the odontoblast markers collagen I, DSPP and DMP1, as well as a decreased level of p-AKT1. Treatment with SC79, an activator of AKT that was able to restore the reduced p-AKT1 caused by the combined knockdown of *Sema3e* and *Col11a2*, failed to restore the protein levels of SEMA3E and COL11A2, indicating that AKT1 is positioned downstream of SEMA3E and COL11A2 (Fig. 6C). Activation of AKT signaling using SC79 significantly rescued the reduced levels of odontoblast markers attributed to the concurrent knockdown of *Sema3e* and *Col11a2*, suggesting that AKT1 mediates the regulation of odontoblast differentiation by SEMA3E and COL11A2 (Fig. 6C). Collectively, these findings indicate that SEMA3E and COL11A2 promote odontoblast differentiation by activating AKT1.

### AKT1 activation partially rescues odontoblast differentiation defects caused by *Setd2* suppression or gene ablation

**Fig. 7.**
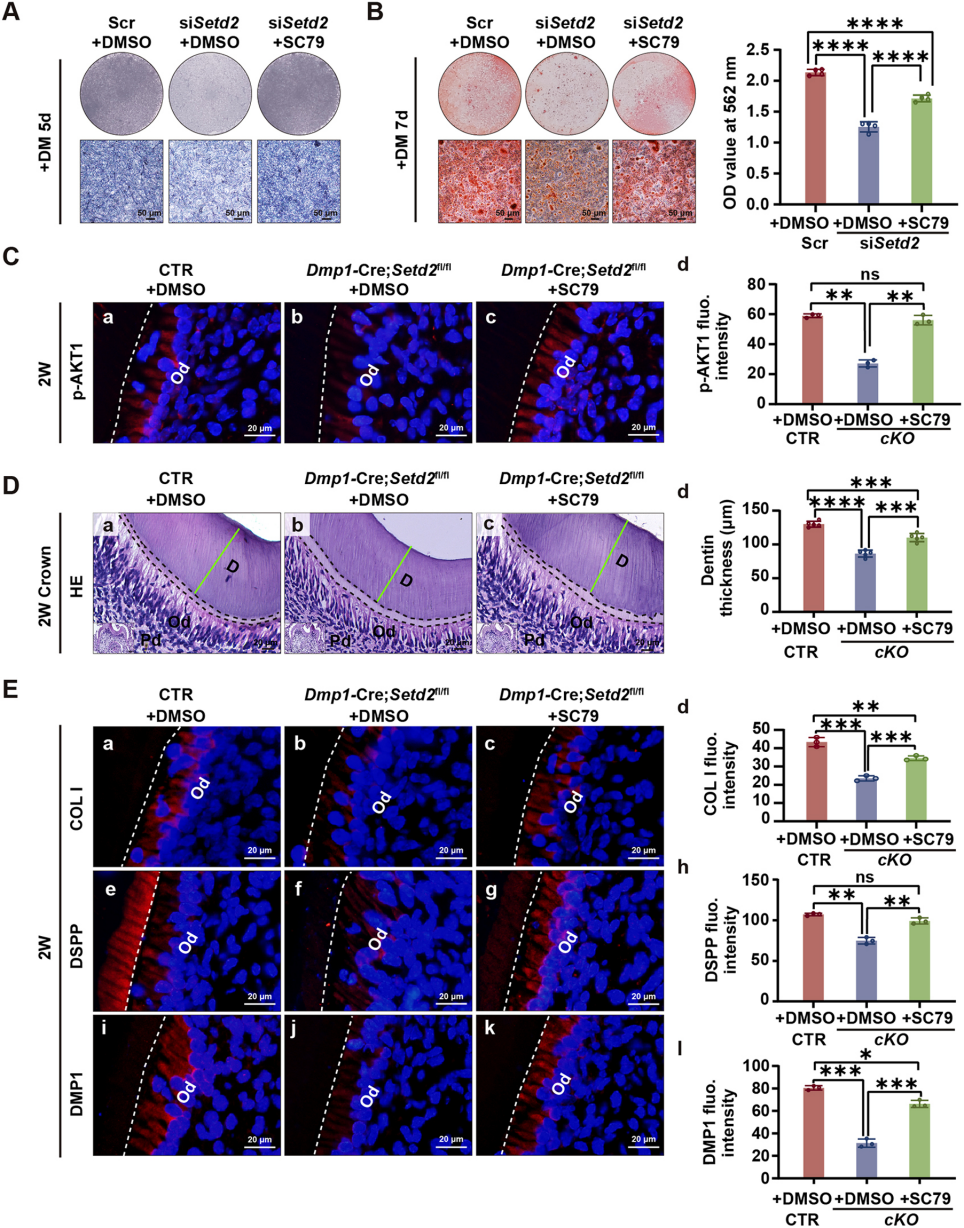
**Activation of AKT1 partially rescues odontoblast differentiation defects caused by *Setd2* knockdown or ablation.** (A) ALP activity was measured in mDPCs cultured with differentiation medium (DM) for 5 days following transfection with scramble siRNA or *Setd2* siRNAs and treatment with or without SC79 (*n*=3). (B) ARS staining was performed to assess the ability of mineralized nodule formation in mDPCs cultured with DM for 9 days following indicated treatment. Stained cells were washed with 10% cetylpyridinium chloride, and the extracted dye was quantified by measuring the optical density (OD) values at 562 nm. The quantification data are presented as mean±s.d. and were analyzed by two-tailed unpaired Student's *t*-test (*n*=3). (C) IF and the quantitative analysis of fluorescence intensity of p-AKT1 in the odontoblast layer of mouse molars. The quantification data are presented as mean±s.d. and were analyzed by two-tailed unpaired Student's *t*-test (*n*=3). (D) HE staining of the mouse molars showing longer cellular processes of odontoblasts and thicker dentin widths (green lines) in *Dmp1*-Cre*;Setd2*^fl/fl^ mice injected with SC79 compared to *Dmp1*-Cre*;Setd2*^fl/fl^ mice at 2W (a-c). Quantitative analysis of dentin thickness in control mice, *Dmp1*-Cre*;Setd2*^fl/fl^ mice, and *Dmp1*-Cre*;Setd2*^fl/fl^ mice injected with SC79 at 2W (d). The quantification data are presented as mean±s.d. and were analyzed by two-tailed unpaired Student's *t*-test (*n*=5/group). (E) IF and the quantitative analysis of fluorescence intensity of odontoblast markers (collagen I, DSPP and DMP1) in the odontoblast layer of mouse molars at 2W. The quantification data are presented as mean±s.d. and were analyzed by two-tailed unpaired Student's *t*-test (*n*=3). Dashed lines in C and E delineate the front of predentin contacting with the odontoblasts. CTR, control mice; D, dentin; Od, odontoblasts; Pd, predentin; Scr, scramble.*P<0.05; **P<0.01; ***P<0.001; ****P<0.0001.

To examine further whether AKT1 is the key mediator of the facilitating role of SETD2 on odontoblast differentiation, we conducted *in vitro* and *in vivo* rescue experiments using SC79. Western blot analysis revealed that treatment with SC79 significantly rescued the reduced levels of the odontoblast markers collagen I, DMP1 and DSPP caused by *Setd2* knockdown (Fig. 6C). In cultured mDPCs, ALP and ARS staining showed that the decreased ALP activity and mineralization ability due to *Setd2* knockdown were rescued by SC79 treatment (Fig. 7A,B).

To investigate the *in vivo* rescue effects, *Dmp1*-Cre*;Setd2*^fl/fl^ mice were injected with SC79 on P3, P6 and P12, and sacrificed at 2W. AKT1 was successfully activated, as evidenced by increased expression of p-AKT1 tested by IF (Fig. 7C). HE staining revealed that injection of SC79 rescued the reduced widths of dentin in both the crown and root regions in *Dmp1*-Cre*;Setd2*^fl/fl^ mice at 2W (Fig. 7D, Fig. S8). Furthermore, reduced expression of collagen I, DSPP and DMP1 due to *Setd2* deletion were also rescued by SC79 administration (Fig. 7E).

## DISCUSSION

SETD2 plays a pivotal role in orchestrating the growth and development of diverse tissues and organs by modulating cell behaviors. SETD2 has been identified as a crucial regulator of the fate of BMSCs, with its deficiency leading to bone loss and marrow adiposity (Wang et al., 2018). Recently, it has been discovered that pancreatic deletion of *Setd2* causes abnormalities in both exocrine and endocrine lineages, leading to abnormal pancreas development in mouse (Lu et al., 2024). In our study, we demonstrate that SETD2 is expressed during odontoblast differentiation. Loss of SETD2 reduces both H3K36me3 occupancy on the loci of *Col11a2* and *Sema3e* and their expression levels. The reduced expression of COL11A2 and SEMA3E leads to impaired AKT1 activation and diminished odontoblast differentiation and dentin formation.

SETD2 is known to be able to trimethylate H3K36 within chromatin (Michalak et al., 2019). We analyzed the expression and distribution of SETD2 and H3K36me3 and found similar patterns between them during odontoblast differentiation. SETD2 was observed in the nucleus of odontoblasts. This subcellular localization is consistent with its role as an HMT.

The function of SETD2 in odontoblast differentiation was investigated through several experiments. Initially, gene knockdown experiments revealed that SETD2 promotes the odontoblastic differentiation of mDPCs. Subsequently, to investigate the *in vivo* function of SETD2, we generated a conditional knockout mouse model, *Ubc-*CreERT2*;Setd2*^fl/fl^. We primarily analyzed the phenotype of the first mandibular molars in mice, because, compared with mouse incisors, the developmental process of mouse molars is more similar to that of human teeth (Tummers and Thesleff, 2003). *Ubc-*CreERT2*;Setd2*^fl/fl^ mice displayed impaired odontoblast differentiation characterized by shorter cell processes, and impaired dentinogenesis characterized by reduced dentin widths, as well as increased proportions of non-mineralized predentin relative to total dentin. A previous study has shown that the interaction between odontoblasts and vascular endothelial cells in the dental pulp may influence dentin formation (Matsubara et al., 2022). Therefore, the ubiquitous deletion of *Setd2* in *Ubc-*CreERT2*;Setd2*^fl/fl^ mice after tamoxifen injection cannot exclude the potential influence of other adjacent cell types on odontoblasts. Next, we generated *Dmp1*-Cre*;Setd2*^fl/fl^ mice to delete *Setd2* specifically in the odontoblast layer. These mice demonstrated thinner dentin as well as decreased expression of collagen I, DSPP and DMP1 in molars. An increase in the quantity or proportion of predentin indicates that the balance between secretion and mineralization has not been well maintained. *Dmp1*-Cre*;Setd2*^fl/fl^ mice exhibited an increased proportion of non-mineralized predentin relative to total dentin. Therefore, these findings underscore the significant contribution of SETD2 to dentinogenesis. Similarly, SETD2 was previously found to facilitate the differentiation of BMSCs into osteoblasts (Wang et al., 2018).

In our study, we used siRNA to knock down *Setd2* in mDPCs and induced their differentiation into odontoblast-like cells. To investigate the genome-wide impact of *Setd2* knockdown on H3K36me3 distribution, we performed spike-in CUT&Tag analysis targeting H3K36me3. Our sequencing results revealed H3K36me3 enrichment at the promoter and gene body regions. The robust enrichment of H3K36me3 at the promoters is consistent with a recent study (Zhang et al., 2022). In contrast, when traditional ChIP-seq was performed, H3K36me3 signals were mainly enriched in the gene body regions (Zhang et al., 2022). This discrepancy may be attributed to chromatin disruption during ChIP-seq procedures, whereas the CUT&Tag technique avoids crosslinking and sonication, and preserves native chromatin, thereby capturing these signals effectively (Zhang et al., 2022). SMYD5 is known to catalyze H4K20me3 (Kidder et al., 2017). It has been recently identified to catalyze H3K36me3 on gene promoters (Zhang et al., 2022). Additionally, SETD5, which shows weak H3K9me1 methyltransferase activity *in vitro* (Pinheiro et al., 2012), has been reported to catalyze H3K36me3 in neural stem cells (Sessa et al., 2019). Our analysis of previously published scRNA-seq data (Jing et al., 2022) revealed that *Setd5* exhibited notable expression in mouse molars, including in odontoblasts, and *Smyd5* also showed detectable expression in odontoblasts (Fig. S9B). These findings imply complicated regulation of H3K36me3 modification in teeth, with other HMTs possibly compensating for H3K36me3 after *Setd2* deletion.

Integrated analysis of RNA-seq and spike-in CUT&Tag-seq data revealed that *Setd2* deficiency reduces both H3K36me3 occupancy on the loci of and the mRNA levels of *Col11a2* and *Sema3e*, suggesting that SETD2-mediated H3K36me3 may play a role in facilitating their transcription. Reduced protein levels of COL11A2 and SEMA3E in the odontoblasts of *Dmp1*-Cre*;Setd2*^fl/fl^ mice and in the odontoblastic cells derived from *Dmp1*-Cre*;Setd2*^fl/fl^ mice supported the positive regulation of their expression by SETD2. COL11A2 and SEMA3E have been reported to play important roles in the development of other organs or systems. COL11A2 is associated with several skeletal dysplasia diseases, including fibrochondrogenesis (Tompson et al., 2012) and Stickler syndrome, which is characterized by cartilage destabilization and abnormal skeletal shape and properties (Avcin et al., 2008; Li et al., 2001; Melkoniemi et al., 2000). *Sema3e*^−/−^ mice show abnormal development of the branched aortic plexus with a much narrower avascular midline (Meadows et al., 2012). Our research has for the first time revealed that COL11A2 and SEMA3E promote odontoblastic differentiation of mDPCs. Prior studies have shown that COL11A2 and SEMA3E are involved in the activation of the PI3K/AKT signaling (Agrawal et al., 2024; Hagihara et al., 2022; Wang et al., 2023). It has been reported that the PI3K/AKT signaling is activated in the inner enamel epithelium of miniature pigs at E50, and by E60 PI3K-Akt signaling has transitioned to the odontoblast layer and promotes the differentiation of odontoblasts (Wang et al., 2023). The *in vitro* and *in vivo* experiments in this study showed that administration of SC79, an activator of AKT activity, partially rescued the defects in the odontoblast differentiation and dentin formation caused by *Setd2* knockdown or ablation, or caused by knockdown of *Col11a2* and *Sema3e*. Thus, SETD2 regulates the expression of COL11A2 and SEMA3E, which activate AKT1 signaling pathway to maintain odontoblast differentiation. However, in pancreatic cancer cells, *Setd2* ablation results in sustained AKT activation through enhanced intrinsic extracellular matrix production, ultimately promoting tumor metastasis (Niu et al., 2020). This study, combined with our findings, revealed that although the AKT pathway acts as a common downstream target of SETD2 in both cell types, its activation levels and associated biological functions are highly context dependent. Therefore, the role of SETD2 in modulating AKT signaling exhibits remarkable cell-type specificity. Our study found that both COL11A2 and SEMA3E play important roles in the activation of AKT1. However, it remains unclear whether these two proteins regulate AKT1 activation through the same or distinct receptors and signaling pathways. A previous study showed that SEMA3E activated the PI3K/AKT pathway via its receptor PLXND1 (Hagihara et al., 2022). We analyzed scRNA-seq data (Jing et al., 2022) and observed the expression of *Plxnd1* in the odontoblasts of mouse molars at P3.5 (Fig. S9C). This suggests that SEMA3E might activate intracellular signaling pathway by binding to PLXND1 in odontoblasts. COL11A2, along with COL11A1 and COL11A3, forms collagen type XI, a component of the extracellular matrix. It has been reported that collagen type XI binds to integrin α2β1 and discoidin domain receptor tyrosine kinases 2 (DDR2) in cancer-associated fibroblasts, inhibiting cancer-associated fibroblast-mediated collagen remodeling and tumor cell invasion (Zeltz et al., 2022). We observed the transcripts of *Ddr2*, *Itga2* and *Itgb1* in the odontoblasts of mouse molars at P3.5 by analyzing published scRNA-seq data (Fig. S9D) (Jing et al., 2022). Therefore, DDR2 and integrin α2β1 might act as receptors for COL11A2 on the odontoblast membrane, which needs further experiments for verification. Meanwhile, further studies are needed to clarify whether these pathways cooperate or act independently.

SETD2-mediated H3K36me3 has been found to play roles in fine-tuning transcription, alternative splicing, mRNA methylation and DNA mismatch repair in mammalian cells (Huang et al., 2019; Neri et al., 2017; Wagner and Carpenter, 2012; Yuan et al., 2017). The interactions of H3K36me3 with the associated reader protein(s) determine its impact on cells (Lam et al., 2022). For example, in mammalian cells, morf related gene 15 (MRG15; MORF4L1) binds to H3K36me3 to regulate exon definition and splicing, and SETD2-mediated H3K36me3 influences alternative splicing of genes such as *Fgfr2* and *Pkm2* (*Pkm*) through MRG15-dependent polypyrimidine tract-binding protein (PTB; PTBP1) recruitment (Luco et al., 2010). SETD2-mediated H3K36me3 inhibits pluripotency and promotes the differentiation of mouse embryonic stem cells at least in part through METTL14-dependent regulation of m^6^A modification on several crucial pluripotency genes, such as *Oct4* (*Pou5f1*), *Nanog* and *Sox2* (Huang et al., 2019). Therefore, the effects of H3K36me3 modification vary across cell types due to its linking to different co-factors in distinct cell contexts. The intimate mechanism(s) and co-factors through which SETD2-mediated H3K36me3 modulates the expression of *Col11a2* and *Sema3e* need further investigation.

SETD2 also methylates nonhistone proteins, including α-tubulin, STAT1 and EZH2 (Chen et al., 2017; Park et al., 2016; Yuan et al., 2020). It is evident that, due to the widespread nature of histone modifications and their functional diversity, SETD2-mediated H3K36me3 exerts its regulatory effects on multiple biological processes rather than being limited to a single gene or signaling pathway. Our study reveals a part of the broader changes induced by SETD2 loss in odontoblasts. Whether SETD2 also regulates odontoblast differentiation through methylation of certain nonhistone substrates needs to be investigated in the future.

**Fig. 8.**
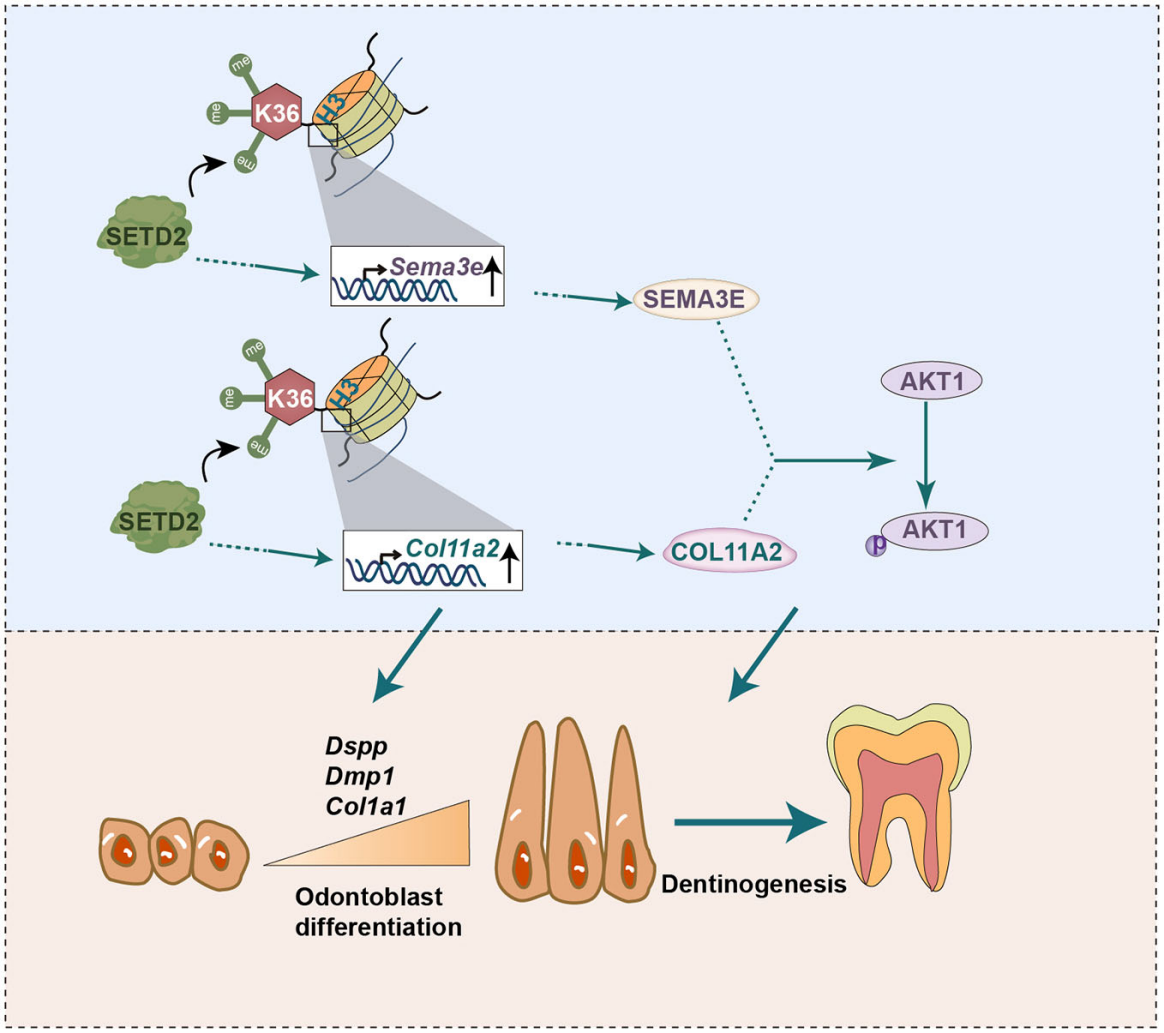
**Schematic illustrating the role of SETD2 in regulating odontoblast differentiation and dentinogenesis.** SETD2 catalyzes the trimethylation of H3K36. SETD2-mediated H3K36me3 is correlated with the expression of *Col11a2* and *Sema3e*, upregulation of which facilitates AKT1 activation, promoting odontoblast differentiation and dentin formation.

Taken together, we show that SETD2 is highly expressed in the nucleus during odontoblast differentiation. The expression of *Col11a2* and *Sema3e* was found to be correlated with changes in H3K36me3 levels, which are influenced by SETD2. Furthermore, we prove that COL11A2 and SEMA3E exert a role in activating AKT1, thereby regulating odontoblast differentiation and dentinogenesis (Fig. 8).

## MATERIALS AND METHODS

### Animals

Mice were maintained in compliance with the requirements of protocols approved by the Animal Care and Ethical Committee of Wuhan University (WP20210576). All mice were born and maintained under pathogen-free conditions at ∼20-24°C with a humidity of ∼40-70% and a 12/12-h dark/light cycle (lights on at 07.00 h, lights off at 19.00 h), with free access to water and food (Center for Animal Experiment, Wuhan University). We acquired *Dmp1*-Cre*;Setd2*^fl/fl^ mice by crossing *Setd2*^fl/fl^ mice with *Dmp1*-Cre mice, and obtained *Ubc-*CreERT2*;Setd2*^fl/fl^ mice by crossing *Setd2*^fl/fl^ mice with *Ubc-*CreERT2 mice. For CreER activation, tamoxifen was dissolved in corn oil. Mice were injected at a concentration of 20 mg/kg at P4, P6, P10 and P12, and sacrificed at P14. The mice were generated on the C57BL/6J background and were genotyped by PCR. Sequences are listed in Table S5. Same-sex mice were used for comparison. *Setd2*^fl/fl^ mice were used as the control. Detailed information about the animal samples is given in Table S6.

### Tissue isolation and cell culture

Primary mDPCs were isolated as previously described (Fu et al., 2021). Briefly, mouse dental papillae were separated from the first mandibular molars of mice at E16.5 and seeded onto cell plates after digestion with 0.25% trypsin for 10 min. mDPCs were cultured in Dulbecco's modified Eagle's medium (DMEM; HyClone) supplemented with 10% fetal bovine serum (Gibco) and 100 U/ml of penicillin/streptomycin (HyClone) at 37°C with 5% CO_2_. For induction of odontoblastic differentiation, cells were cultured in DMEM containing 10% fetal bovine serum, 10 mM sodium β-glycerophosphate (Sigma-Aldrich), 50 mg/ml L-ascorbic acid (Sigma-Aldrich) and 100 nM dexamethasone (Sigma-Aldrich) The medium was changed every other day.

### Cell transfection and pharmacological treatments

siRNAs were designed and synthesized by GenePharma. Three interference sequences were designed for the target gene. The interference efficiency was verified by RT-qPCR. Two siRNAs with knockdown efficiencies of >50% were selected for further experiments. Sequences are listed in Table S7. mDPCs were seeded onto plates and transfected with siRNAs with Lipofectamine 3000 (Invitrogen) according to the manufacturer's instructions.

SC79 (MedChemExpress) was dissolved in dimethyl sulfoxide. mDPCs were treated with 10 μM SC79 after siRNA transfection. For the *in vivo* rescue experiments, SC79 was dissolved in corn oil. Following gene identification, mice were injected at a concentration of 10 mg/kg at P3, P6 and P12, and sacrificed at P14.

### Calcein-ARS double labeling

P3 mice were injected intraperitoneally with 20 mg/kg Calcein (2.5 mg/ml in 2% NaHCO_3_ solution) and 40 mg/kg ARS (2 mg/ml in H_2_O) at P3 and P10 separately. Mice were euthanized at P14, and the mandibles were fixed. Then, mandibles were dehydrated, and embedded by Servicebio. The samples were sectioned at 5 μm with a hard tissue cutter. Slices were observed under an Aperio digital Pathology microscope (Leica).

### μ-CT

The collected mandibles were fixed in 4% polyoxymethylene for 1 day and then stored in 70% ethanol at 4°C before processing. The mandibles were scanned on a Perkin Elmer quantum GX2 instrument (Perkin Elmer). Images were acquired at an effective pixel size of 10 μm, voltage of 50 kV, current of 114 μA and exposure time of 14 min for each of the 360 rotational steps. CTAn software was used to reconstruct two-dimensional image slices. Quantitative analyses were performed using Mimics Research 20.0 software.

### ALP staining and ARS staining

mDPCs cultured with growth or differentiation medium were fixed with 4% paraformaldehyde (PFA). The BCIP/NBT Alkaline Phosphatase Kit (Beyotime) was used following the standard procedure for ALP staining.

For ARS staining, cells were stained with 1% ARS solution (pH 4.5) made of ARS powder (Aladdin) and ddH_2_O. The stained cells were then dissolved in 10% cetylpyridinium chloride at 37°C overnight. The quantitative values of the solution were measured under an absorbance of 562 nm.

### RT-qPCR

Total RNAs were extracted from mDPCs using Trizol reagent (Takara Bio), and cDNA was synthesized from 1 μg RNA using the ABScript III RT Master Mix (Abclonal) for qPCR with gDNA Remover (Abclonal). RT-qPCR was performed using the ChamQ SYBR qPCR Master Mix (Yeason) under the Bio-Rad CFX RT-PCR system. Primers are listed in Table S8.

### HE staining, IHC and IF staining of tissues and cells

Tissues were isolated as previously described (Zheng et al., 2020). Dissected mouse mandibles at different developmental stages were fixed in 4% PFA for 1 day at 4°C, and decalcified in 10% EDTA. Mandibles were then dehydrated, embedded in paraffin, and sliced into 5-μm-thick sections.

For HE staining, slides were deparaffinized, rehydrated, and stained with Hematoxylin and Eosin dyes.

For IHC, antigen retrieval of the slices was performed using 10 mmol/l citrate buffer (pH 6.0). Slices were then incubated using the HRP Polymer anti-Rabbit IHC Kit (MaxVision) according to the manufacturer's instructions. Anti-SETD2 (Cell Signaling Technology) and anti-H3K36me3 antibodies (see Table S9) were applied at a 1:200 concentration diluted with PBS. After staining with diaminobenzidine, nuclei were counterstained with Hematoxylin. Then, the slices were mounted onto glass slides using neutral balsam, and covered with coverslips.

For IF, tissue slides were blocked with 3% bovine serum albumin for 1 h at 37°C and incubated with each primary antibody at 4°C overnight. The concentrations of primary antibodies applied were as follows: DMP1 (1:200; Abclonal), DSPP (1:200; Novus Biologicals), p-AKT1 (1:200; Immunoway), SEMA3E (1:200; R&D Systems) and COL11A2 (1:200; Immunoway) (see Table S9). Then, Alexa Fluor Red 594 Donkey Anti-Rabbit IgG (1:300; ANT030s, Antgene) or Alexa Fluor Green 488 Donkey Anti-Goat IgG (1:300; ANT025s, Antgene) was used as the secondary antibody. Then, the slices were mounted onto glass slides with antifade mounting medium with DAPI (P0131, Beyotime), and covered with coverslips.

For cell IF, mDPCs were fixed with 4% PFA and permeabilized with 0.1% Triton X-100 in PBS. Cells were then treated as for tissue IF.

### Western blotting

NP-40 (Beyotime) containing 1/100 protease inhibitor cocktail (MedChemExpress) and phosphatase inhibitor (MedChemExpress) was added to lyse the cells for 15 min at 4°C. After supersonic lysis and centrifugation (12,000 ***g*** for 10 min), the supernatant was obtained. Then, the protein samples were mixed with 20% SDS and denatured at 95°C for 15 min. Protein lysates were separated by SDS-polyacrylamide gel electrophoresis and transferred to polyvinylidene difluoride membranes (Millipore). After blocking with 5% skim milk in TBS and 0.1% Tween 20, membranes were incubated with the desired primary antibodies overnight at 4°C. Then, membranes were washed and incubated with the appropriate secondary antibodies (goat anti-rabbit-HRP, 1:5000, AP13P, Sigma-Aldrich; or goat anti-mouse-HRP, 1:5000, Ap124p, Sigma-Aldrich) at room temperature for 1 h. Signals were detected by Clarity Western ECL Substrate (BIO-RAD). The primary antibodies were as follows: DSPP (1:1000; Novus Biologicals), DMP1 (1:1000; Abclonal), β-actin (1:10,000; Abclonal), H3 (1:10,000; AntGene), H3K36me3 (1:1000; Abcam), SEMA3E (1:1000; R&D Systems), COL11A2 (1:500; Immunoway), AKT1 (1:1000; Immunoway), p-AKT1 (1:1000; Immunoway), SETD2 (1:1000; LSBio), vinculin (1:1000; Immunoway) and collagen I (1:1000; Proteintech). Details of the antibodies are given in Table S9.

### Laser-capture microdissection

All reagents were RNase-free. Under RNase-free and sterile condition, mouse mandibles at P3 were isolated and fixed in 4% PFA for 1 day at 4°C. Then, the mandibles were decalcified in 10% ethylenediaminetetraacetic acid (EDTA) for ∼7-10 days. Next, the mandibles were infiltrated with 10%, 20% and 30% sucrose to dehydrate, embedded in OCT (TissueTek), and snap-frozen using liquid nitrogen. Tissues were sectioned at 8 μm and placed on membrane slides (MMI) for laser microdissection. Odontoblasts were laser-captured using laser-capture microdissection (MMI) and allowed into a sterile collecting isolation tube cap (MMI) containing 50 μl buffer RLT (79216, QIAGEN). Laser-capture microscopy-procured odontoblasts from an average of 20-25 sections were pooled from multiple mandibles. Total RNAs were extracted using the RNeasy Micro Kit (QIAGEN), and samples were treated with DNase I to remove genomic DNA contamination. Extracted RNA quality and quantity were evaluated using a NanoDrop 2000c Spectrophotometer (Thermo Fisher Scientific).

### Spike-in CUT&Tag

mDPCs were transfected with *Setd2* siRNAs or scramble siRNA and cultured for 5 days under the odontoblastic induction condition. CUT&Tag was performed as described previously (Kaya-Okur et al., 2019), with modifications using the Hyperactive Universal CUT&Tag Assay Kit for Illumina Pro (Vazyme, TD904). In brief, samples containing 1×10^5^ cells each were collected. Then, the nuclei were isolated and incubated with Concanavalin A-coated magnetic beads at room temperature for 10 min. After removal of the supernatant, the nuclei-bound beads were resuspended in 50 μl antibody buffer containing 1 μg rabbit anti-H3K36me3 antibody (ab9050, Abcam) or rabbit nonimmune IgG (A7016, Beyotime) and incubated at 4°C overnight. The beads were then sequentially incubated with a goat anti-rabbit secondary antibody (ab6702, Abcam) and pA/G-Transposome Mix, and underwent fragmentation. Following the manufacturer's instructions, 1 pg of spike-in DNA was added to each sample. The spike-in DNA sequence is listed in Table S10. The reaction was terminated, and DNA fragments were extracted using phenol-chloroform-isoamyl alcohol. For CUT&Tag-seq, the DNA fragments were PCR-amplified using indexed P5 and P7 primers. The library products were then enriched, quantified and sequenced using the Illumina NovaSeq 6000 platform in PE150 mode.

### CUT&Tag-qPCR

qPCR was performed using specific primers for spike-in DNA, *Col11a2* and *Sema3e*. The primer sequences for CUT&Tag-qPCR assays are listed in Table S10. The 2^−ΔΔC^_T_ method was used for data analysis as follows: (1) ΔC_T_ value for each group was calculated as ΔC_T treatment_=C_T treatment_−C_T treatment spike-in DNA_ for the experimental group, ΔC_T control_=C_T control_−C_T control spike-in DNA_ for the control group, or ΔC_T IgG_=C_T IgG_−C_T IgG spike-in DNA_ for the negative control (IgG); (2) 2^−ΔΔC^_T_ value for each group was calculated with IgG group as the reference as relative enrichment fold_treatment_=2^−(ΔC^_T treatment_^−ΔC^_T IgG_^)^ for the experimental group, relative enrichment fold_control_=2^−(ΔC^_T control_^−ΔC^_T IgG_^)^ for the control group, or relative enrichment fold_IgG_=2^−(ΔC^_T IgG_^−ΔC^_T IgG_^)^=1 for the negative control.

### CUT&Tag-seq data processing

High-throughput sequencing was conducted by Anoroad in Beijing, and the raw data can be downloaded from GSE288311. The raw sequencing data underwent quality control using Skewer (version 0.2.2) to filter out low-quality reads, and adapter contamination was trimmed using FastQC (version 0.11.5). The filtered data were then aligned to the spike-in genome using Bowtie2 (version 2.2.9) with the following parameters: -p 8--very-sensitive-local --no-unal --no-mixed --no-discordant -- phred33 -l 10 -X 700 -x spike_in_genome.fa. Clean reads were then aligned to the *Mus musculus* reference genome (GRCm39) using BWA (version 0.7.12-r1039) with parameters -T 25 -k 18. DeepTools (version 3.0.2) was used to visualize the distribution of reads spanning 5 kb upstream of the transcription start site (TSS) to 5 kb downstream of the transcription end site. MACS (version 2.1.2) was used for peak calling. The parameters were -p 0.05 --call-summits --nomodel --shift −100 --extsize 200 --keep-dup all. The peaks were annotated to the nearest genes based on proximity to the closest TSS, and the resulting gene list was used for downstream analyses. Decreased binding peaks were defined by filtering for entries with log_2_ fold change ≤−1, *P*<0.05 and fold enrichment >2. The corresponding genes and their GO annotations are listed in Table S11. Detailed information regarding all software and their parameters is listed in Table S12.

### RNA-seq and data processing

mDPCs were isolated from control mice and *Dmp1*-Cre*;Setd2*^fl/fl^ mice at P6. Total RNAs were extracted from mDPCs using Trizol reagent (Takara Bio) and then subjected to PE150 HiSeq, which was performed by Anoroad in Wuhan. Each sample contained pooled RNAs from three biological replicates and was mixed with an equal mass of RNAs to minimize variation across samples. After the samples were qualified, ∼1-3 μg total RNAs of each sample was used as the starting material to construct a transcriptome sequencing library. The raw data can be downloaded as GSE288310.

Bowtie2 was used for building the genome index, and the clean data were then aligned to the reference genome using HISAT2 (version 2.2.1). Transcript assembly was performed using StringTie (version 2.2.1) on the aligned RNA-seq reads. Fragments per kilobase millon mapped (FPKM) reads for each gene in each sample were counted using HTSeq (version 2.0.2), and FPKM was then calculated to estimate the expression level of genes in each sample. DEGseq2 (version 1.40.2) was used for differential gene expression analysis. Genes with *P*<0.05, q<1 and |log_2_Foldchange|≥0.58 were identified as differentially expressed genes. GO analysis and KEGG analysis were performed using Database for Annotation, Visualization and Integrated Discovery (https://david.ncifcrf.gov). To provide a visual representation of the data, an online platform for data analysis and visualization, https://www.bioinformatics.com.cn (last accessed on 20 February 2024), was used. The specific software and parameters used for analysis are listed in Table S13.

### Analysis of scRNA-seq

scRNA-seq data were obtained from Gene Expression Omnibus (GSE189381) (Jing et al., 2022). A joint graph was laid out in 2D using the uniform manifold approximation and projection method through CONOS routine embed Graph with parameters spread=1 and min. Dist=0.05. The graph-based Leiden community method with resolution=1.0 was used to partition cells into five clusters.

### Statistical analysis

Statistical analyses of all data were performed with GraphPad Prism 8 and data are presented as mean±s.d. Comparisons were performed using a two-tailed Student's *t*-test or one-way analysis of variance (ANOVA). *P*<0.05 was defined as statistically significant.

## Supplementary Material

10.1242/develop.204352_sup1 Supplementary information

Table S1. The Gene Ontology (GO) enrichment terms based on RNA-seq data from dental mesenchymal cells of *Dmp1*-Cre;*Setd2*^fl/fl^ mice compared to controls. Upregulated and downregulated genes were analyzed separately.

Table S2. The Kyoto Encyclopedia of Genes and Genomes (KEGG) pathway enrichment terms based on RNA-seq data from dental mesenchymal cells of *Dmp1*-Cre;*Setd2*^fl/fl^ mice compared to controls. Upregulated and downregulated genes were analyzed separately.

Table S3. The 310 overlapped genes with differential expression levels and reduced H3K36me3 occupancy via integration of the RNA-seq and spike-in CUT&Tag-seq data. Differentially expressed genes were obtained using RNA-seq data of dental mesenchymal cells from *Dmp1*-Cre;*Setd2*^fl/fl^ mice versus those from control mice. Genes with reduced H3K36me3 occupacy were obtained using Spike-in CUT&Tag-seq targeting H3K36me3 in odontoblastlike cells transfected with si*Setd2* compared with those transfected with scramble siRNA.

Table S4. Gene Ontology (GO) enrichment analysis conducted using the 310 overlapped genes identified from integration of RNA-seq and spike-in CUT&Tag-seq datasets.

Table S11. Genes with significantly decreased H3K36me3 CUT&Tag peaks were annotated. Peaks were defined by log_2_ fold change ≤,-1, p < 0.05, and fold enrichment > 2.
