# Supplementary material for: H3K36me3 modification by SETD2 is essential for Col11a2 and Sema3e transcription to maintain dentinogenesis in mice
Source: Development. 2025 Jul 14;152(14):dev204352. doi: 10.1242/dev.204352 (PMC12338916; doi:10.1242/dev.204352)
Supplement: Supplementary information [file develop-152-204352-s1.pdf]

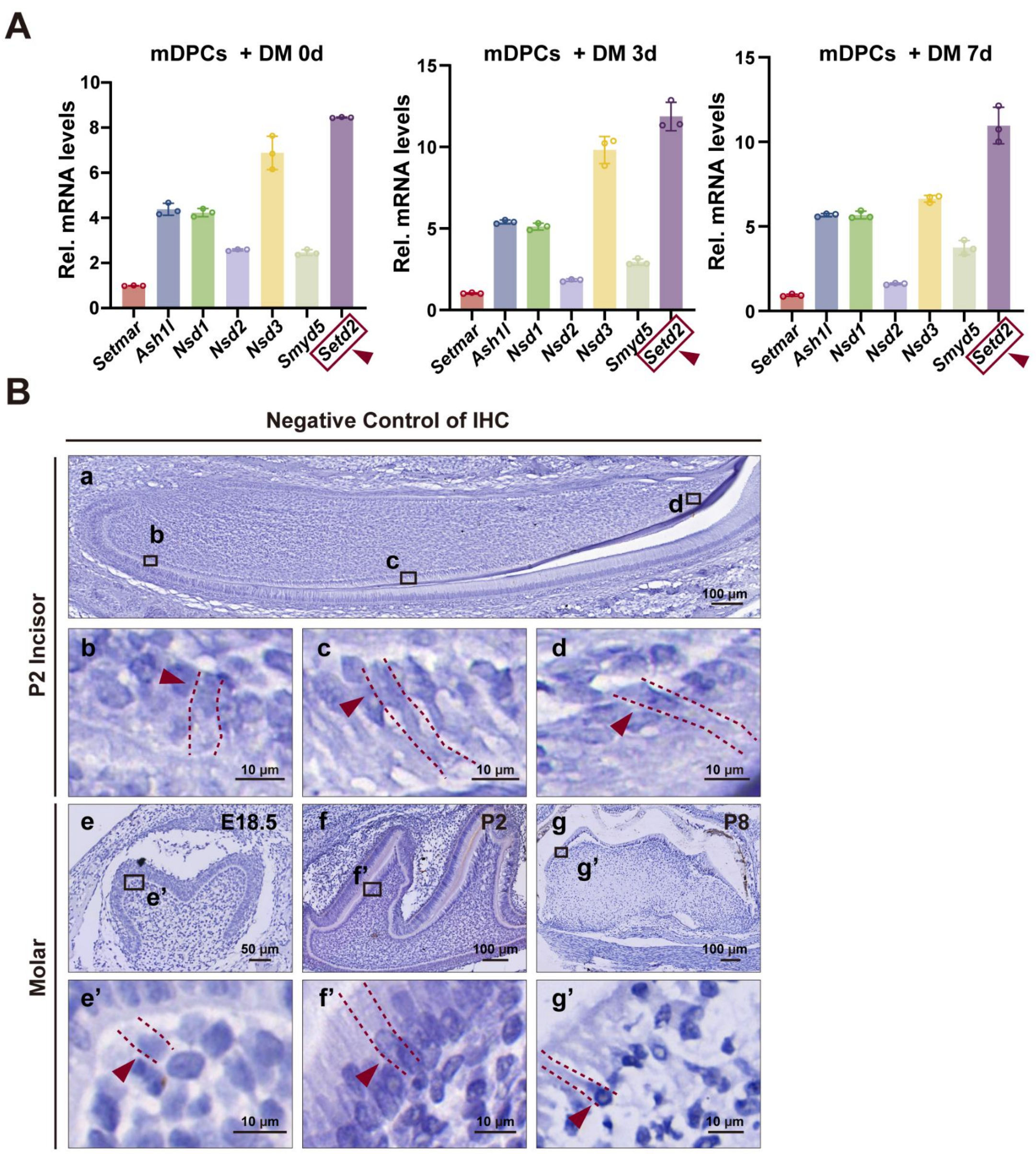

Fig. S1. The mRNA levels of H3K36 methyltransferases during the odontoblastic differentiation process of mDPCs and the negative control for IHC.

(A) The mRNA levels of H3K36 methyltransferases were assessed by RT-qPCR during the odontoblastic differentiation process of mDPCs. **(B)** The negative control of IHC. The normal nonimmune IgG was used as the primary antibody in mouse incisors at P2 (a-d) and molars at E18.5 (e, e'), P2 (f, f'), and P8 (g, g'). The magnified views depicted in (e'), (f'), and (g') correspond to the rectangles in (e), (f), and (g), respectively. (P2, *n*=6; E18.5, *n*=3; P8, *n*=3). mDPCs, mouse dental papilla cells; E, embryonic day; P, postnatal day.

Development • Supplementary information

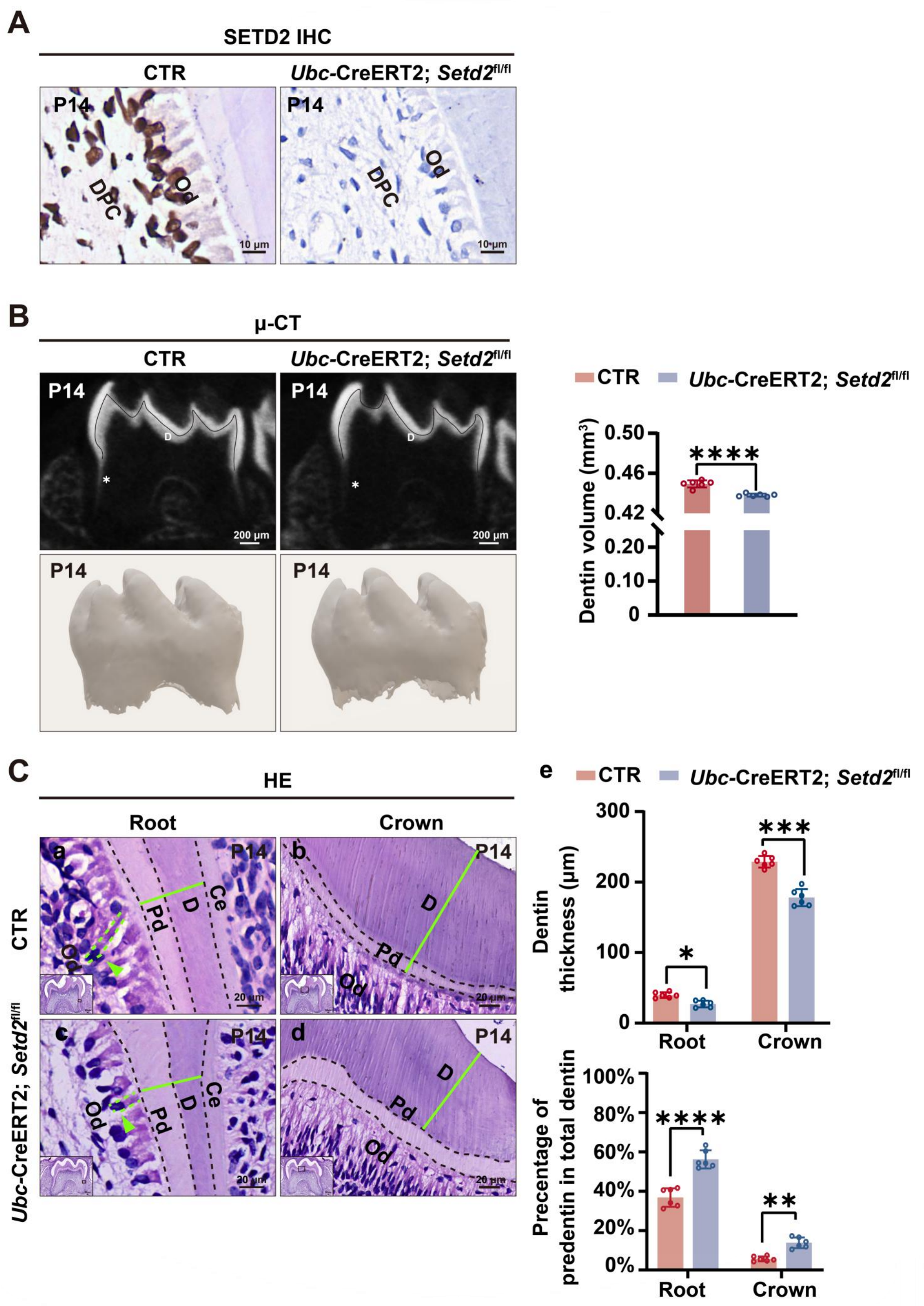

Fig. S2. The dentin phenotype in *Ubc*-CreERT2;*Setd2*$^{fl/fl}$ mice. (A) The expression of SETD2 is absent in the mouse molars from *Ubc*-CreERT2;*Setd2*$^{fl/fl}$ mice but present in those from the control (CTR) mice shown by IHC. **(B)** The μ-CT images of the first mandibular molars show decreased dentin widths in *Ubc*-CreERT2; *Setd2*$^{fl/fl}$ mice compared to control mice at P14. Quantitative analysis after three-dimensional reconstruction demonstrated reduced dentin volume

Development • Supplementary information

in *Ubc*-CreERT2;*Setd2*$^{fl/fl}$ mice at P14. The quantification data are presented as mean ± s.d. and analyzed by two-tailed unpaired Student's t-test (P14, *n*=6/genotype). **(C)** HE staining of the mouse molars shows shorter cellular processes of odontoblasts and thinner dentin thickness in *Ubc*-CreERT2; *Setd2*$^{fl/fl}$ mice (c, d) compared with control mice (a, b) at P14. (e) The dentin thickness as well as the percentage of predentin in total dentin were quantified and compared between control and *Ubc*-CreERT2;*Setd2*$^{fl/fl}$ mice at P14. The thickness of predentin and dentin were measured in one section every six consecutive sections of six HE-stained samples. The quantification data are presented as mean ± s.d. and analyzed by two-tailed unpaired Student's t-test (*n*=6/genotype). D, dentin; Pd, predentin; Od, odontoblasts; CTR, control mice; P, Postnatal day; ns, not significant; *P<0.05; **P<0.01; ***P<0.001; ****P<0.0001.

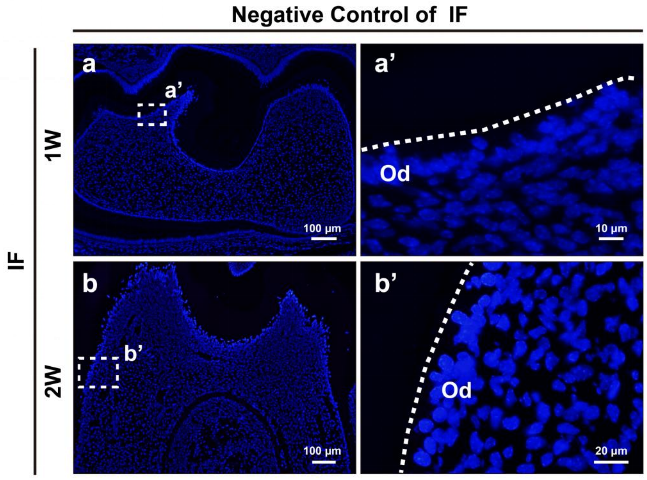

Fig. S3. The negative control for IF. (a) Negative control for IF of mouse molars at 1W. (b) Negative control for IF of mouse molars at 2W. (a') and (b') respectively depict enlarged views of the regions outlined by the white boxes in (a) and (b). Od, odontoblasts.

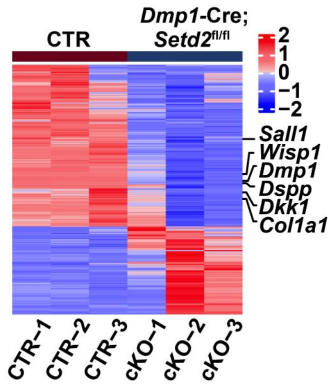

Fig. S4. Heat map of up-and down-regulated genes in the dental papilla cells of *Dmp1*-Cre*;Setd2*$^{fl/fl}$ mice compared with control mice (*n*=3).

Development • Supplementary information

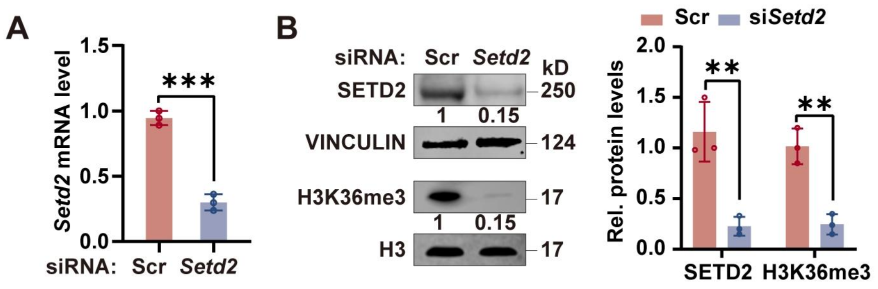

Fig. S5. The knockdown efficiency of *Setd2* siRNA. (A) *Setd2* mRNA level was assessed using RT-qPCR in the mDPCs cultured with differentiation medium (DM) for 5 days following transfection with the scramble or *Setd2* siRNA. The quantification data are presented as mean ± s.d. and analyzed by two-tailed unpaired Student's t-test (*n*=3). **(B)** The levels of SETD2 and H3K36me3 were measured by Western blot in the mDPCs cultured with DM for 5 days following transfection with the scramble or *Setd2* siRNA. The protein levels of SETD2 and H3K36me3 were calculated using VINCULIN and Histone H3 as the loading control respectively. The quantification data are presented as mean ± s.d. and analyzed by two-tailed unpaired Student's t-test (*n*=3). Scr, scramble.

Development • Supplementary information

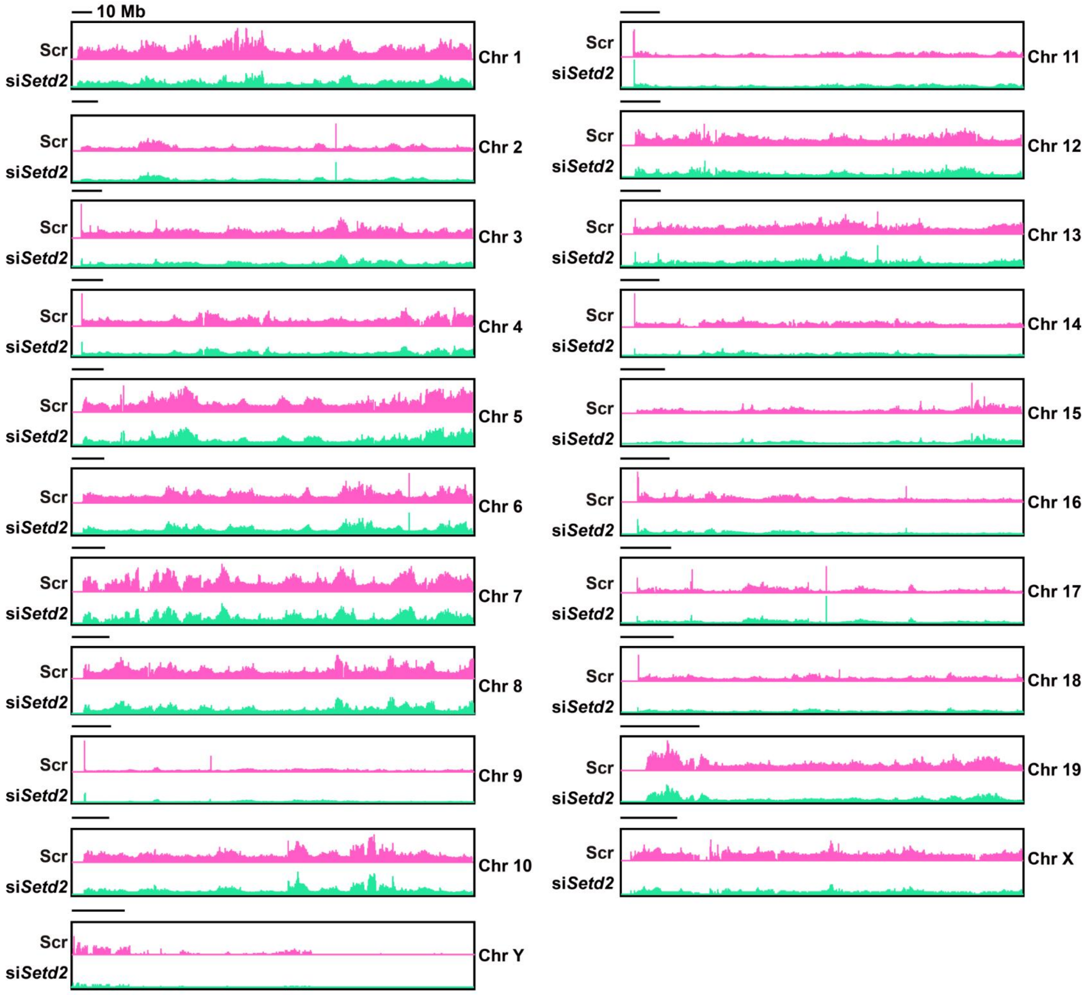

Fig. S6. H3K36me3 CUT&Tag-seq profiles of 19+X/Y chromosomes of the odontoblast-like cells with *Setd2* knockdown compared to control cells. The black bars on top of the panels represent the 10-mb scale. All panels have the same signal scale of 0–5 RPM on the y-axis.

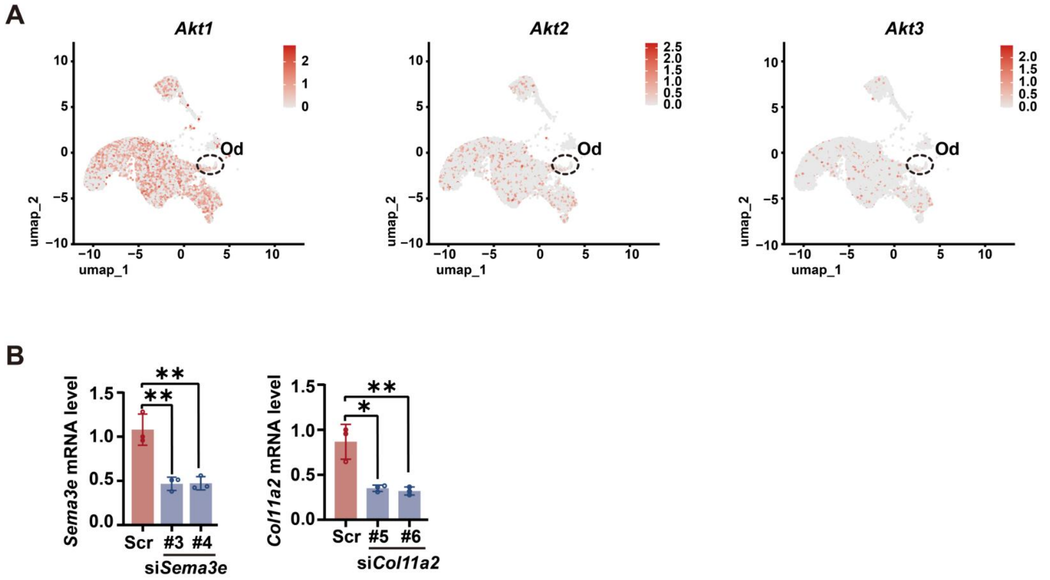

Fig. S7. Expression of AKT isoforms in the odontoblasts and validation of *Sema3e* and *Col11a2* siRNA knockdown efficiency.

(A) The transcripts for *Akt1, Akt2* and *Akt3* in molars were analyzed using UMAP from the dataset (GSE189381). **(B)** The knockdown efficiency of *Sema3e* and *Col11a2* siRNA in mDPCs detected by RT-qPCR. The quantification data are presented as mean ± s.d. and analyzed by one-way ANOVA with Tukey's post-test (*n*=3). Scr, scramble; Od, odontoblasts.

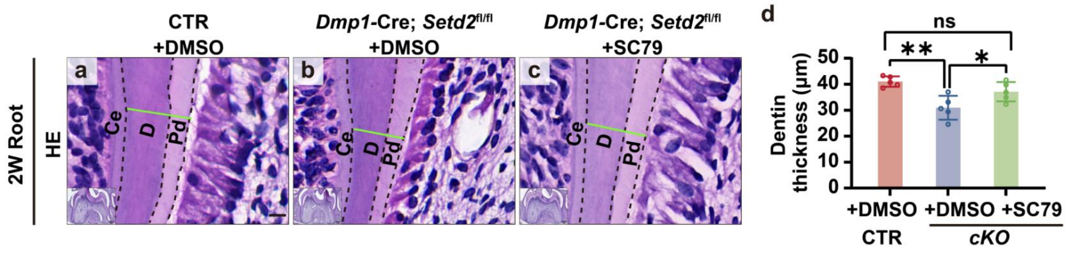

Fig. S8. HE staining of the mouse molars in control mice, *DmSp1*-Cre;*Setd2*$^{fl/fl}$ mice, and *Dmp1*-Cre;*Setd2*$^{fl/fl}$ mice injected with SC79 at 2W. HE staining of the mouse molars shows longer cellular processes of odontoblasts and thicker dentin widths in *Dmp1*-Cre;*Setd2*$^{fl/fl}$ mice injected with SC79 compared to *Dmp1*-Cre;*Setd2*$^{fl/fl}$ mice at 2W (a-c). Quantitative analysis of the dentin thickness in control mice, *Dmp1*-Cre;*Setd2*$^{fl/fl}$ mice, and *Dmp1*-Cre;*Setd2*$^{fl/fl}$ mice injected with SC79 at 2W is shown in (d). The thickness of dentin in roots was measured in one section every three consecutive sections of five HE-stained samples. The quantification data are presented as mean±s.d. and analyzed by two-tailed unpaired Student's t-test (*n*=5/group).

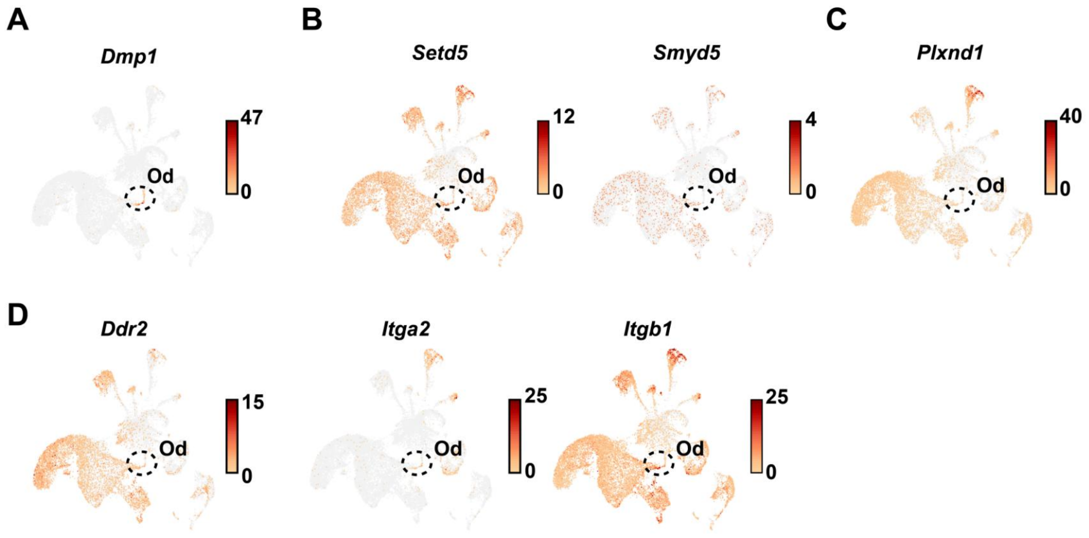

Fig. S9. Expression of *Dmp1*, *Setd5, Smyd5, Plxnd1, Ddr2, Itga2,* and *Itgb1* in molars. UMAP plots using the dataset (GSE189381) illustrated the transcripts for *Dmp1* **(A)**, *Setd5*, *Smyd5* **(B)**, *Plxnd1* **(C)**, *Ddr2*, *Itga2* and *Itgb1* **(D)**. Od, odontoblasts. Dashed circles indicate the odontoblast cluster.

Table S1. The Gene Ontology (GO) enrichment terms based on RNA-seq data from dental mesenchymal cells of *Dmp1*-Cre*;Setd2*$^{fl/fl}$ mice compared to controls. Upregulated and downregulated genes were analyzed separately.

Available for download at
https://journals.biologists.com/dev/article-lookup/doi/10.1242/dev.204352#supplementary-data

Table S2. The Kyoto Encyclopedia of Genes and Genomes (KEGG) pathway enrichment terms based on RNA-seq data from dental mesenchymal cells of *Dmp1*- Cre*;Setd2*$^{fl/fl}$ mice compared to controls. Upregulated and downregulated genes were analyzed separately.

Available for download at
https://journals.biologists.com/dev/article-lookup/doi/10.1242/dev.204352#supplementary-data

Development • Supplementary information

Table S3. The 310 overlapped genes with differential expression levels and reduced H3K36me3 occupancy via integration of the RNA-seq and spike-in CUT&Tag-seq data. Differentially expressed genes were obtained using RNA-seq data of dental mesenchymal cells from *Dmp1*-Cre;*Setd2*$^{fl/fl}$ mice versus those from control mice. Genes with reduced H3K36me3 occupancy were obtained using Spike-in CUT&Tag- seq targeting H3K36me3 in odontoblast-like cells transfected with si*Setd2* compared with those transfected with scramble siRNA.

Available for download at
https://journals.biologists.com/dev/article-lookup/doi/10.1242/dev.204352#supplementary-data

Table S4. Gene Ontology (GO) enrichment analysis conducted using the 310 overlapped genes identified from integration of RNA-seq and spike-in CUT&Tag-seq datasets.

Available for download at
https://journals.biologists.com/dev/article-lookup/doi/10.1242/dev.204352#supplementary-data

Table S5. Primer sequences for genotyping of mice.

Genotype	**Forward（5’-3’）**	**Reverse（5’-3’）**
***Setd2*$^{fl/fl}$**	TGTTTGTGTTGACAATGAGC	TGATGCAAATTCATGAGGTA
***Dmp1*-Cre**	ATGCCCACCAAAGTCATCAGT GTAG	AGCTGCACCATCAACATGCC
***Ubc*-CreERT2**	GACGTCACCCGTTCTGTTG	CTAGGCCACAGAATTGAAAGA TCT
	GTAGGTGGAAATTCTAGCATC ATCC	AGGCAAATTTTGGTGTACGG

Table S6. Summary of the mice used in this study.

Genotype	Age	Experiments	Sample number	Total number
Kunming mice	E16.5	Primary mDPCs isolation	200 (The total number of mouse embryos used for cell experiments)	200
Kunming mice	E18.5, P2, P7, P8	Histological analysis	3 for each age	12
Setd2$^{fl/fl}$ (Control)	E16.5	Western blot analysis	30	30
	P12, P14, 24W	Micro-CT	6 for each age	18
	1W, 2W, 3W	Histological analysis	5 for each age	15
	P14	Calcein-alizarin red S double labeling	5	5
	PN6	Dental papilla collection for RNA-Seq	6	6
	PN3	Laser Capture Microdissection	10	10
	P14	DMSO	5	5
Dmp1-Cre;*Setd2*$^{fl/fl}$	E16.5	Western blot analysis	30	30
	P12, 24W	Micro-CT	6 for each age	12
	1W,	Histological analysis	5 for each	15

Development • Supplementary information

	2W, 3W		age	
	P14	Calcein-alizarin red S double labeling	5	5
	PN6	Dental papilla collection for RNA-Seq	6	6
	P14	DMSO treatment	5	5
	P14	SC79 treatment	5	5
Ubc-CreERT2;*Setd2*$^{fl/fl}$	P14	Micro-CT	6	6
	P14	Histological analysis	6	6

Table S7. siRNA sequences used in this study.

siRNA	Forward（5'-3'）	Reverse（5'-3'）
Scramble siRNA	UUCUCCGAACGUGUCACGUTT	ACGUGACACGUUCGGAGAATT
siSetd2#1	GCCUGAAUCCUUACCGGAATT	UUCCGGUAAGGAUUCAGGCTT
siSetd2#2	GCAAGUACCAGAUUCUCUATT	UAGAGAAUCUGGUACUUGCTT
siSetd2#3	GGAAGUCAUUCUAGAGGAATT	UUCCUCUAGAAUGACUUCCTT
siSema3e#1	GCUACGCCUGUCACAUAAATT	UUUAUGUGACAGGCGUAGCTT
siSema3e#2	GCAAAGUAAACGGAGGCAATT	UUGCCUCCGUUUACUUUGCTT
siSema3e#3	GCAGUAAAGGUAGAAGAAUTT	AUUCUUCUACCUUUACUGCTT
siCol11a2#1	GAGGCUUUCCCAAAGACUUTT	AAGUCUUUGGGAAAGCCUCTT
siCol11a2#2	GCAGGGUUCUACGAUUACATT	UGUAAUCGUAGAACCCUGCTT
siCol11a2#3	GAAGGAACCAGCAAGACUUTT	AAGUCUUGCUGGUUCCUUCTT

Table S8. Primer sequences used in this study for RT-qPCR.

Gene	Forward（5'-3'）	Reverse（5'-3'）
Setd2	AGACTGCTGTTCCTCAGTTAAGT	CTGTATCCATTTCCGTGCTCG
Gapdh	TGTGTCCGTCGTGGATCTGA	TTGCTGTTGAAGTCGCAGGAG
Dspp	GTGGGATCATCAGCCAGTCAG	TGCCTTTGTTGGGACCTTCA
Dmp1	ACCACAATACTGAATCTGAAAGCTC	TGCTGTCCGTGTGGTCACTA
Col1	GCTCCTCTTAGGGGCCACT	CCACGTCTCACCATTGGGG

Development • Supplementary information

Phex	GAAAGGGGACCAACCGAGG	AACTTAGGAGACCTTGACTCA CT
Creb3l1	GCCCTGGGAAACAAGCTGT	AGCTGAGTCATTTCTCCTGGG
Wnt11	GCTGGCACTGTCCAAGACTC	CTCCCGTGTACCTCTCTCCA
Enpp1	CTGGTTTTGTCAGTATGTGTGCT	CTCACCGCACCTGAATTTGTT
Sema3e	AGGCTACGCCTGTCACATAAA	CCGTTCTTGATACTCATCCAG C
Col11a2	GAAGGGTGCTCGTGGGAAA	GAGGGCCTGGGTATCCTAGAG
Smyd5	GGCACCCCCTCAATAAGCTG	ACCCAGTGGTCCTTGTCCTT
Setmar	GTAGCCCCGACAGAACAACAA	GTGTGGGGTCAATGTCTGCTC
Ash1l	CCTCGGTGGACTAAAGTGGTG	CGCTGGCTCAGAACTATTTGA
Nsd1	GTTTGGGAAAAGGTAGACCTCAC	TTGTATGGCTCGTTTTGGCAG
Nsd2	TGCCAAAAAGGAG TACGTGTG	CTTCGGGAAAGTCCAAGGCAG
Nsd3	TCCACTGGTGTTAAGTTCCAGG	GGCACCTCTTGTGTTAA TTTTGG

Table S9. Antibodies used in this study.

Antibody specificity	Species	Catalog#	Source
**SETD2**	Rabbit	89680	Cell Signaling Technology
**SETD2**	Rabbit	LS-C332416	LSBio
**H3K36me3**	Rabbit	ab9050	Abcam
**H3**	Mouse	ANT330	AntGene
**DSPP**	Rabbit	NBP2-92546	Novus
**DMP1**	Rabbit	A16832	Abclonal
**SEMA3E**	Goat	AF3239	R&D System
**COL11A2**	Rabbit	YT1009	Immunoway
**AKT1**	Rabbit	A20799	Abclonal
**p-AKT1**	Rabbit	YP0006	Immunoway
**VINCULIN**	Rabbit	YT4882	Immunoway
**β-ACTIN**	Rabbit	6600-1-Ig	Proteintech

Table S10. Primer sequences for CUT&Tag-qPCR and sequence of the spike-in DNA.

Gene	Forward（5'-3'）	Reverse（5'-3'）
Sema3e	AGGCTACGCCTGTCACATAA	TGCACTTTGAGGGTCTTGGG
Col11a2	CACTCCGGGTGGCTATAAGG	CCAACCCAAATTGCCCTGTC
Spike-in DNA	GCCTTCTTCCCATTTCTGATCC	CACGAATCAGCGGTAAAGGT
**Spike-in DNA sequence**	ATAACTCAATGTTGGCCTGTATAGCTTCAGTGATTGCGATTCGCCT GTCTCTGCCTAATCCAAACTCTTTACCCGTCCTTGGGTCCCTGTAG CAGTAATATCCATTGTTTCTTATATAAAGGTTAGGGGGTAAATCCC GGCGCTCATGACTTCGCCTTCTTCCCATTTCTGATCCTCTTCAAAA GGCCACCTGTTACTGGTCGATTTAAGTCAACCTTTACCGCTGATTC GTGGAACAGATACTCTCTTCCATCCTTAACCGGAGGTGGGAATATC CTGCATTCCCGA ACCCATCGACGA.	

Table S11. Genes with significantly decreased H3K36me3 CUT&Tag peaks were annotated. Peaks were defined by $\log_2$ fold change ≤ –1, $p < 0.05$, and fold enrichment > 2.

Available for download at
https://journals.biologists.com/dev/article-lookup/doi/10.1242/dev.204352#supplementary-data

Development • Supplementary information

Table S12. Analysis software for CUT&Tag.

Analysis	Software	Version	Parameters	Description
Trimming	Skewer	0.2.2	-m pe	Filter out the adaptor and low quality reads
Raw data quality control	FastQC	v0.11.5	Default Parameter	
Alignment	Bowtie2	version 2.2.9	-p 8--very-sensitive-local --no-unal --no-mixed --no-discordant --phred33 -I 10 -X 700 -x spike_in_genome.fa	Aligning reads to spike-in sequence
Mapping	BWA	0.7.12-r1039	-T 25 -k 18	Mapping the reads to the genome
Inter-sample correlation	deepTools	3.0.2	--corMethod pearson	
Peak calling	MACS2	2.1.2	-p 0.05 --call-summits --nomodel --shift -100 --extsize 200 --keep-dup all	Gives robust and high resolution peak predictions

Development • Supplementary information

Table S13. Analysis software for RNA-seq.

Analysis	Software	Version	Parameters	Description
Alignment	HISAT2	2.2.1	--dta –t –p 4	Mapping reads to genome
Transcript assembly	StringTie	2.2.1	-G ref.gtf	Sequence assembly
Gene Expression Quantity	HTSeq	2.0.2	-i gene_id -f bam -s no -a 10 -q	Estimating gene expression level
Differential Expression	DESeq2	1.40.2	method='per-condition'	Differentially Expressed Genes Analysis

Development • Supplementary information
